# Effects of Grape By-Products on Oxidative Stress and Inflammation in Farm Animals: An Overview of Studies Performed in Pigs, Chickens, and Cattle

**DOI:** 10.3390/ani15111536

**Published:** 2025-05-23

**Authors:** Klaus Eder, Robert Ringseis, Denise K. Gessner

**Affiliations:** 1 Institute of Animal Nutrition and Nutrition Physiology, Justus Liebig Universität Giessen, Heinrich-Buff-Ring 26-32, 35392 Giessen, Germany; robert.ringseis@ernaehrung.uni-giessen.de (R.R.); denise.gessner@ernaehrung.uni-giessen.de (D.K.G.); 2 Center of Sustainable Food Systems, Justus Liebig Universität Giessen, Senkenbergstrasse 3, 35390 Giessen, Germany

**Keywords:** polyphenols, reactive oxygen species, antioxidant system, farm animals

## Abstract

High-yielding farm animals often face severe metabolic stress, compounded by environmental stressors such as psychosocial stress, heat stress, intensive housing systems, and poor hygiene management. These factors result in oxidative stress and inflammatory processes, which adversely affect both animal health and performance. Polyphenols are known to alleviate both oxidative stress and inflammatory responses. Since grapes are rich in polyphenols, by-products of winemaking could have beneficial effects on these processes. This review aims to provide an overview of the potential antioxidative and anti-inflammatory effects of grape by-products in farm animals. The first section of the review examines the causes and consequences of oxidative stress and inflammation. The second section highlights the general effects of polyphenols in addressing these issues. The third and central part of the review presents an overview of findings from studies investigating the impact of various grape-derived polyphenols on the antioxidant system and inflammation in pigs, chicken, and cattle. Overall, these studies demonstrate that grape by-products can effectively reduce oxidative stress and inflammation in pigs and chickens, often leading to improved performance. In cattle, however, fewer studies have been conducted, and the results regarding oxidative stress and inflammation are less consistent. In conclusion, grape by-products represent valuable feed options for preventing oxidative stress and inflammation in monogastric farm animals (pigs, chickens).

## Simple Summary

In farm animals, high performance and exposure to various stress factors can lead to oxidative stress and inflammation, both of which negatively affect their health and productivity. To address these challenges, it is crucial to develop feeding strategies that can mitigate oxidative stress and inflammation. Winemaking by-products, such as grape pomace and grape seeds, are rich in polyphenols—compounds known for their ability to reduce both oxidative stress and inflammatory responses. This review evaluates the hypothesis, based on a literature analysis, that feeding grape by-products to farm animals (pigs, chickens, and cattle) can help combat these processes. The findings indicate that grape by-products are effective in reducing oxidative stress and inflammation in pigs and chickens. However, the effects in cattle are less consistent and require further investigation. In conclusion, grape by-products prove to be particularly beneficial as feed for monogastric farm animals, not only in preventing oxidative stress and inflammation but also in enhancing their overall well-being.

## 1. Introduction

High-performing animals often experience metabolic stress due to increased feed intake and high productivity demands, which are frequently compounded by environmental factors such as poor housing, heat stress, and suboptimal hygiene. These stress factors can also trigger oxidative stress or inflammation [1,2,3,4]. As detailed in Section 2.1 and Section 2.2 of this review, both oxidative stress and inflammation negatively affect animal health and livestock performance.

Since the EU banned feed antibiotics in 2006, there has been an intensive search for feed additives for farm animals that can maintain or even improve animal health and performance. Natural compounds produced by plants might be relevant candidates in this regard. Plants produce a great variety of secondary metabolites, many of which have been shown to exert a broad range of beneficial effects on health, particularly in humans and rodent models, but also in various farm animal species [5,6,7,8,9]. Among the vast number of secondary plant metabolites, the polyphenol group might be the most promising due to its well-established antioxidant and anti-inflammatory properties [7,10,11,12].

Grapes are especially rich in polyphenols [13,14,15]. During grape processing for winemaking, large amounts of by-products, such as grape pomace, are generated, which can also be used as components in the feed rations of farm animals [16,17]. Many studies have investigated the hypothesis that, due to their high polyphenol contents, grape by-products from winemaking can counteract oxidative stress and inflammatory processes in various farm animal species. However, the results regarding their effects on the antioxidant system and inflammation are not entirely consistent. Moreover, an overview presenting comparative species data on these effects has not yet been published.

Therefore, the aim of this review is to provide a comprehensive overview of current research on the effects of grape by-products as feed additives in pigs, chickens, and cattle, with a particular focus on their influence on oxidative stress and inflammation. In the introductory sections, the generation and role of oxidative stress and inflammation, as well as their effects on animal health and performance, are described. The main section then focuses on studies examining the impacts of grape by-products on oxidative stress and inflammation in pigs, chickens, and cattle.

## 2. Theoretical Background: Oxidative Stress, Inflammation, and Their Interlinkage, and Potential Effects of Polyphenols

### 2.1. Oxidative Stress: Role of Oxidants and Antioxidants

Oxidative stress occurs when there is an imbalance between the production of oxidants in cells or tissues and the antioxidant system, which is responsible for their elimination [18]. When oxidants overwhelm the antioxidant defense system, those that are not neutralized and removed can cause cellular damage. In the body, certain target molecules, including lipids containing polyunsaturated fatty acids (e.g., membrane phospholipids), DNA, proteins, and specific carbohydrates such as hyaluronic acid, are highly susceptible to damage by excessively produced oxidants. Excessive oxidative modification of these molecules leads to cell death via apoptosis or necrosis, as well as structural tissue damage, which can contribute to the development of several diseases in animals, such as pneumonia or enteritis in pigs, mastitis or pneumonia in ruminants, or airway obstructions in horses [19]. In dairy cows, the production of excessive oxidants also leads to dysfunctional immune responses [20,21]. The oxidation of proteins due to oxidation stress has been suggested to affect female reproduction, gut health, and mammary gland function in dairy cows [22].

Oxidants include both radical and non-radical compounds containing oxygen, nitrogen, or chlorine [23]. The most important radicals are superoxide and hydroxyl radicals. Superoxide radicals are primarily formed during electron transport when molecular oxygen gains one electron [24,25]. Other key sources of superoxide radicals are activated neutrophils and macrophages during inflammatory responses, in which these radicals are enzymatically produced by NADPH oxidase [26,27]. Superoxide radicals can interact with other molecules to form additional reactive oxygen species (ROS). A notable reaction in this respect involves superoxide dismutase (SOD), which, within the mitochondrial inner membrane, catalyzes the conversion of superoxide radicals into hydrogen peroxide, another significant type of ROS. Transition metals such as iron and copper in their free form can then react with hydrogen peroxide to produce even more reactive hydroxyl radicals (via the “Fenton Reaction”) [28,29,30]. Several dietary factors may also increase oxidant generation within the body. Dietary polyunsaturated fatty acids incorporated into cellular membranes are susceptible to oxidation, which can be prevented by vitamin E. Therefore, an increased intake of polyunsaturated fatty acids leads to an increased requirement for vitamin E. It has been suggested that an additional 0.5 mg of vitamin E (RRR-α-tocopherol) is required per gram of linoleic acid in humans, and there is even an additional requirement of 1.25 or 1.5 mg of vitamin E per g of eicosapentaenoic or docosahexaenoic acid [31]. Diets with high levels of polyunsaturated fatty acids (such as diets with soybean oil, linseed oil, or fish oil) lead to a reduction in tissue tocopherol concentrations in comparison to diets with low levels of polyunsaturated fatty acids due to an increased turnover of vitamin E [32,33,34]. For farm animals, quantitative data on the additional requirement for vitamin E depending on the intake of polyunsaturated fatty acids are not available. However, it seems plausible that there is a similar increase in required vitamin E due to the increased uptake of polyunsaturated fatty acids in animals. Elevated levels of lipid peroxidation products in plasma or tissues, indicative of oxidative stress, are especially observed when a high intake of polyunsaturated or oxidized fatty acids is combined with insufficient vitamin E [33,35,36]. Other dietary factors, such as environmental pollutants (e.g., pesticides and organic solvents) or mycotoxins (e.g., aflatoxins), stimulate the generation of oxidants. These substances induce the production of ROS by activating the xenobiotic system in the liver [37,38]. The xenobiotic system, a metabolic system located within the endoplasmic reticulum (ER), is responsible for the biotransformation and elimination of foreign compounds (xenobiotics) in the body. It consists of two phases. In phase I, xenobiotics undergo enzymatic reactions that introduce reactive or polar groups, mainly by cytochrome P450 oxidases. In phase II, these modified compounds are conjugated to polar compounds (such as glucuronic acid or sulfate groups). After phase II, xenobiotics are generally less toxic and can be eliminated via bile or urine [39]. However, the oxidation of some chemical compounds via cytochrome P450-catalyzed reactions produces superoxide and other highly reactive compounds that not only induce oxidative stress but also cause cytotoxicity and genotoxicity [40,41].

In contrast to the historical view that oxidants exert deleterious effects in cells exclusively and contribute to the development of diseases, recent studies have shown that physiological levels of oxidants act as important physiological regulators of intracellular pathways. For instance, it has been shown that oxidants are able to modulate the activity of redox-sensitive target proteins by modifying cysteine residues. Therefore, the concentrations of oxidants are regulated in cells at a physiological level [42]. When oxidant concentrations increase in the body, adaptive responses are triggered to counteract cell and tissue damage. This includes activating antioxidant enzymes, chaperones, heme oxygenases, DNA repair systems, and ferritin (which binds free iron ions) [43,44]. A crucial component of this adaptive system is the nuclear factor-erythroid 2-related factor-2 (Nrf2), a transcription factor regulating the expression of antioxidant and cytoprotective genes. In its inactive state, Nrf2 is bound to Keap1, a protein that prevents its translocation into the nucleus. However, ROS can interact with critical cysteine residues in Keap1, leading to its degradation via the ubiquitin–proteasome system (UPS) and activating Nrf2. Active Nrf2 translocates into the nucleus, where it induces the expression of various genes containing antioxidant response elements in their promoters. These genes encode proteins involved in glutathione synthesis and conjugation, antioxidant proteins or enzymes [including SOD, catalase (CAT), glutathione peroxidase (GPx), and glutathione reductase (GR) as the most important], xenobiotic-metabolizing enzymes and transporters, metal-binding proteins (e.g., metallothionein or ferritin), and anti-inflammatory proteins [45,46,47]. These cytoprotective proteins not only mitigate oxidative stress but also enhance xenobiotic metabolism, maintain cellular homeostasis, and support cellular detoxification [43]. Additionally, low-molecular-weight antioxidants, such as ascorbic acid, tocopherols, tocotrienols, carotenoids, uric acid, glutathione, and dietary flavonoids and polyphenols, contribute to the defense against oxidants. These molecules interact with oxidants in a relatively nonspecific manner, providing a protective buffer against oxidative damage [48,49,50]. It has been shown that under specific conditions (e.g., in endurance athletes or in models of caloric restriction), the moderate stimulation of ROS production in mitochondria can induce health-promoting effects or even increase lifespan. This phenomenon, known as mitohormesis [51], is based on the fact that oxidants induce adaptive responses, such as the activation of Nrf2, which aims to protect cells from damage by oxidative stress [52,53], while high doses of antioxidants that prevent these oxidant signals interfere with these health-promoting effects [54,55].

### 2.2. Inflammation: Regulation and Consequences

An inflammatory process is a physiological response of the innate immune system to an injury or a pathogen. It is the first line of defense against pathogens, but it also enables the repair of cell damage and tissue injury [56]. Typical reactions that occur during inflammation include redness, swelling, warmth, and pain. These reactions result from increased blood flow and the increased permeability of blood vessels, which allow leukocytes and large molecules such as antibodies or cytokines to pass from the bloodstream into the damaged tissue. The aim of the inflammatory process is to eliminate invading pathogens or toxins and to restore the damaged tissue [57]. An inflammatory process is triggered by the production of a wide spectrum of cytokines, chemokines, adhesion molecules, eicosanoids, or complement proteins [58]. These molecules form a complex network that stimulates blood flow to the damaged tissue, thereby facilitating the entry of immune cells. Additionally, immune cells are activated, and systemic responses, such as increases in body temperature and heart rate and a decrease in appetite, are triggered [59]. On a molecular level, the inflammatory process is controlled by nuclear factor kappa B (NF-κB), the key transcriptional regulator in both innate and adaptive immunity. Among other factors, NF-κB can be activated by oxidative stress. Therefore, there is a direct link between oxidative stress and inflammation. Viruses, bacterial toxins, pro-inflammatory cytokines, and various other stress factors can also activate NF-κB, thereby inducing inflammation. The activation of NF-κB leads to the expression of its target genes, which include pro-inflammatory cytokines such as tumor necrosis factor α (TNF-α), various interleukins (ILs), chemokines, inflammatory enzymes, adhesion molecules, and various receptors [60]. At a physiological level, inflammation is a biological response that aims to identify and eliminate a threat. It is important that the intensity of the inflammation is high enough to fight the infection. However, if inflammation remains uncontrolled, it may progress to chronic inflammation, ultimately leading to self-destruction, which forms the basis of inflammatory diseases [56,61].

A secondary reaction to an inflammatory process is the acute-phase response (APR), which is initiated by synergistically and additively acting endotoxins such as lipopolysaccharides and lipoteichoic acid—classified as pathogen-associated molecular patterns—and various pro-inflammatory cytokines, such as IL-6, IL1β, tumor necrosis factor (TNF)-α, transforming growth factor (TGF)-β, IL8, and IL-22 [62,63]. During the APR, over 200 proteins are produced, primarily in the liver [64]. The acute-phase proteins (APPs) play a crucial role in the systemic response during inflammation, for example, through pathogen opsonization, the scavenging of toxic substances, and the overall regulation of different stages of inflammation [65]. Under healthy conditions, the concentrations of APPs are very low, while their concentrations increase drastically during inflammation [60,64]. Haptoglobin (Hp) and serum amyloid A (SAA) in cattle and Hp, SAA, C-reactive protein, and pig major acute-phase protein in swine are the major proteins [66,67]. Many methodological assays are presently available to measure these parameters and are still being improved to increase their specificity, sensitivity, user-friendliness, and economic availability. In cattle and swine, Hp and SAA are commonly used as biomarkers of inflammation [68]. In cattle, the main applications are the diagnosis and monitoring of frequent diseases such as mastitis and metritis in dairy cows and respiratory problems in young calves. In pigs, APPs are useful in the control of bacterial and viral infections, and they may be used at the slaughterhouse to monitor subclinical pathologies and improve food safety. APPs have even been proposed as biomarkers to assess growth performance since there is an inverse correlation between serum APP concentrations and production parameters. It has been shown that stimulation of the immune system and increased inflammation are accompanied by reduced growth performance due to anorexia, as well as the partitioning of nutrients away from growth to support the immune system [66].

While the concentrations of APPs rise significantly during the early phase of inflammation, the production of other proteins in the liver, such as albumin, apolipoproteins, transferrin, and retinol-binding protein, is noticeably reduced during inflammation. The biological significance of the reduced production of these proteins, referred to as negative APPs, lies in conserving amino acids, which can then be used for the synthesis of APPs or gluconeogenesis, and in restoring homeostasis after stress [65,69,70].

The production of cytokines as a result of an inflammatory reaction is not only crucial for initiating an immune response to pathogens or toxins. Cytokines and other inflammatory mediators produced at the site of inflammation can also enter the brain through the bloodstream, where they can trigger the production and release of cytokines in the mediobasal hypothalamus, inducing a pro-inflammatory state. This, in turn, initiates alterations in neurological activity that influence appetite, body temperature, and metabolic programs regulating body mass and energy homeostasis [71,72]. The changes in metabolic programs caused by pro-inflammatory states in the hypothalamus aim to release energy and amino acids from stores (fat tissue, muscle tissue) and direct them to metabolic pathways that support the immune system. In this context, the breakdown of muscle protein through the activation of the UPS plays a key role. The UPS is stimulated by increased glucocorticoid release, which is a result of hypothalamic–pituitary axis activation during pro-inflammatory conditions [71,73], and by direct activation by pro-inflammatory cytokines [74]. Pro-inflammatory cytokines also suppress the anabolic effects of insulin, thereby inhibiting muscle protein synthesis [75]. The purpose of increased protein breakdown and reduced protein synthesis in the muscle is to conserve amino acids, which are available in the liver for the synthesis of APPs [76,77]. Amino acids released from the muscle are also used for gluconeogenesis. This process is further stimulated by increased cortisol release from the adrenal cortex during inflammation. The glucose resulting from this process is provided to activated immune cells, such as neutrophils and macrophages, which rely on glucose for energy production [78]. The increased release of fatty acids from adipose tissue, which results from the stimulation of lipolysis by elevated catecholamine secretion from the adrenal medulla—a process regulated by the hypothalamus—serves to meet the body’s increased energy demands to raise body temperature (fever generation) and enhance immune system activity [78].

Another important reaction within the framework of inflammation, which is associated with impaired animal performance, is the induction of anorexia [79]. Systemic inflammation leads to the increased production of anorexigenic peptides in the arcuate nucleus of the hypothalamus, while the formation of orexigenic peptides is reduced. Consequently, there is a reduction in appetite, leading to reduced feed intake [71,80]. The loss of appetite in sick animals is believed to have evolved as a survival strategy to fight against pathogen invasion and to facilitate recovery [81,82]. By eating less, animals require less energy and fewer nutrients for anabolic processes, allowing the body to conserve energy and redirect it toward immune system activities, such as combating pathogens and repairing tissues. Many pathogens depend on nutrients from the host’s diet to survive, of which iron is particularly important [83,84]. The optimal iron concentration for the growth of most bacteria is much higher than the concentration freely accessible in the host [85]. Pathogens have thus developed strategies to obtain iron from their host organisms. For instance, bacteria possess several receptors that are able to bind to and take up heme or iron from the host [84]. In turn, host defense mechanisms have been developed that target this dependence to deprive microbes of iron. The reduction in iron availability by reducing iron intake from diet is one strategy in this respect. Other strategies include mechanisms that aim to lower iron absorption in the small intestine by upregulating hepcidin (the master regulator of iron absorption) or the formation of iron-related APPs such as ferritin or lactoferrin (which sequester free iron), ceruloplasmin (which decreases the availability of nonheme iron), haptoglobin (which binds to free hemoglobin), or hemopexin (which binds to free heme). The coordinated action of these proteins can deprive pathogens of iron [83,84,86]. Another important protein built by the host is lipocalin-2, which binds to bacterial siderophores (iron-binding molecules), reducing the availability of iron for bacteria and inhibiting their growth [83].

**Figure 1:**
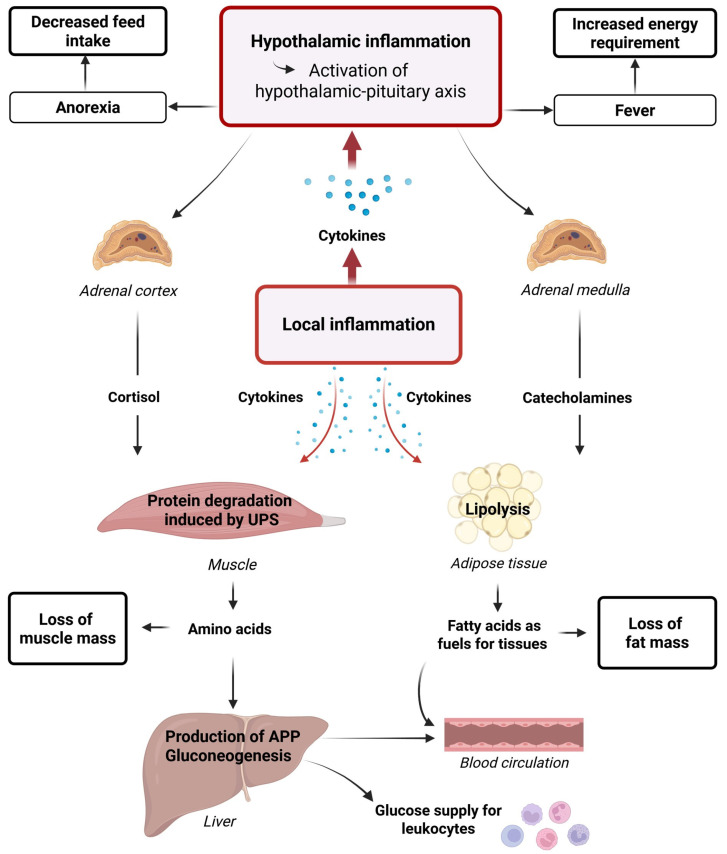
A simplified overview of the hormonal and metabolic changes triggered by inflammation (in reference to [5]). During an inflammatory response, nutrients are released from stores (muscle, adipose tissue) to meet the energetic demands of the inflammation process. Cytokines are released in the affected tissues, which not only act on the local tissue but also stimulate the release of amino acids from the muscle by activation of the ubiquitin–proteasome system (UPS) and fatty acids from adipose tissue by activation of lipolysis. Cytokines can also cause hypothalamic inflammation, which induces anorexia and, through increased release of cortisol or catecholamines, contributes to proteolysis in the muscle and lipolysis in adipose tissue. Amino acids released from the muscle are used for the synthesis of acute-phase proteins (APP) and gluconeogenesis in the liver. Glucose produced by gluconeogenesis is utilized as an energy fuel by activated leukocytes. Fatty acids released are utilized as fuel for tissues, such as for enhanced thermogenesis, leading to the generation of fever. Created in BioRender. Eder, K. (2025) https://BioRender.com/xhvasdt, accessed on 25 April 2025.

A study in infected mice demonstrated that survival positively correlated with anorexia and weight loss, at least in the short term. In contrast, force feeding the infected mice increased their mortality and shortened their survival time [87]. An overview of the hormonal and metabolic changes induced by inflammation is provided in Figure 1.

### 2.3. Link Between Oxidative Stress, Inflammation, and Stress of the Endoplasmic Reticulum

A number of studies have shown that both oxidative stress and inflammation can contribute to the development of endoplasmic reticulum stress (ER stress). ER stress refers to a state in which the folding capacity in the ER is insufficient to adequately fold the whole protein load within the ER lumen. As a result, unfolded or misfolded dysfunctional proteins accumulate in the ER. In turn, an adaptive response is activated, aiming to restore ER homeostasis [88]. This response, known as the unfolded protein response (UPR), triggers three different cellular reactions: (i) the increased production of ER chaperones, such as immunoglobulin heavy-chain binding protein (BiP), to enhance protein folding capacity; (ii) the attenuation of protein translation to reduce the protein load; and (iii) the activation of ER-associated degradation (ERAD), a cellular mechanism that identifies misfolded or defective proteins in the ER and marks them for degradation by the proteasome. This process is essential for protein quality control and helps maintain cellular homeostasis [89,90]. If ER stress becomes excessive and ER homeostasis cannot be restored, the UPR can initiate cellular apoptosis. This action serves to preserve tissue functionality by eliminating dysfunctional cells [91,92]. The UPR is activated when misfolded or unfolded proteins accumulate in the ER. To restore protein homeostasis, the cell initiates a complex signaling cascade regulated by three main transducers, namely, inositol-requiring 1 (IRE1), PKR-like ER kinase, and activating transcription factor 6. Under physiological conditions, when there is no stress, these transducers are bound to the chaperone BiP in the ER lumen, keeping them in an inactive state. When unfolded or misfolded proteins accumulate in the ER lumen, BiP dissociates from the transducers, activating them and triggering the UPR [93]. The activation of PERK stimulates the phosphorylation of eukaryotic initiation factor (eIF) 2α, leading to the attenuation of protein synthesis. The activation of IRE1 results in the activation of the transcription factor X-box binding protein 1 (XBP1), which in turn enhances the production of chaperones and ERAD components and increases phospholipid biosynthesis, causing ER expansion through membrane enlargement. The activation of XBP1 also stimulates the expression of genes involved in ERAD, lipid biosynthesis, ER expansion, and protein folding. The initiation of the UPR also improves cellular defense by activating Nrf2 and triggering inflammation through the upregulation of pro-inflammatory genes. These measures aim to protect the cell from further damage that could exacerbate ER stress. The UPR also activates the release of fibroblast growth factor 21 (FGF21), a hormone that plays an important role in the stress response by providing energy through the stimulation of lipolysis, fatty acid oxidation, gluconeogenesis, or ketogenesis [88,94,95]. To date, most studies on the role of FGF21 have been performed with rodents, while there have been fewer studies in farm animals in this respect. However, there is evidence that FGF21 also plays an important role in stress adaptation in farm animals. In both cows and sows, significantly increased production of FGF21 in the liver has been observed during lactation [96,97,98,99,100,101]. It has been suggested that this process is an adaptation to a negative energy balance, metabolic stress, and ER stress during this phase [102].

ER stress is a well-documented phenomenon in obese individuals. Obesity induces chronic inflammation in adipose tissue, leading to the increased production of ROS and oxidative stress. Both oxidative stress and inflammation stimulate ER stress and activate the UPR. Additionally, the enhanced release of fatty acids—especially saturated fatty acids—from adipose tissue and their uptake by cells in obese individuals promote UPR initiation [103,104]. Such an effect is also present in dairy cows that are over-conditioned during the dry phase. Cows with a high body condition score have a lower feed intake and a more pronounced negative energy balance associated with a greater increase in non-esterified fatty acid mobilization from adipose tissue during early lactation compared to cows with a lower body condition score [105,106,107]. These alterations favor the occurrence of oxidative stress and inflammation, which could also cause ER stress [108,109]. Recent studies suggest that the effect of the enhanced release of fatty acids on the development of ER stress may primarily be caused by alterations in ER membrane composition, associated with reduced membrane fluidity [110]. Numerous studies have demonstrated that ER stress plays a crucial role in the development of pathological conditions or diseases, including non-alcoholic fatty liver disease, insulin resistance, type 2 diabetes, heart disease, cancer, or neurodegeneration in humans [111,112].

In farm livestock, the occurrence of ER stress has been described only to a limited extent. However, there are already some indications that ER stress in the liver may play a role in the occurrence of fatty liver and possibly associated diseases, such as ketosis or insulin resistance in dairy cows [113,114].

### 2.4. Effects of Polyphenols on Oxidative Stress and Inflammation

A large number of studies, conducted either in vitro with cell cultures or in vivo in animal models, show that polyphenols from grapes or grape extracts exhibit antioxidant and anti-inflammatory effects [56,115,116].

The antioxidant properties of polyphenols are based on their chemical structure. Polyphenols contain aromatic rings with hydroxyl groups (-OH), which can donate electrons without becoming unstable themselves. This helps to prevent or reduce oxidative damage by neutralizing oxidants, such as superoxide radicals [117]. Furthermore, polyphenols can activate antioxidative and cytoprotective signaling pathways. The most important of these is the activation of Nrf2. This activation occurs because cells recognize polyphenols as xenobiotic compounds that could potentially be toxic to the cell. According to the so-called hormesis concept, defense systems are thereby activated, with the aim of breaking down and detoxifying polyphenols. Consequently, alongside antioxidative genes, genes in the xenobiotic system (Phase I and Phase II enzymes) are also activated, which not only break down polyphenols but also eliminate ROS and other potentially toxic substances. The activation of defense systems by polyphenols thus leads to an overall enhanced resistance to oxidants and other harmful substances [118,119,120]. A further indirect antioxidant effect of polyphenols is related to their capacity to chelate redox-active metals such as iron and copper, which, in their free (unbound) form, are able to induce the formation of hydroxyl radicals from hydrogen peroxide via the Fenton reaction. Under healthy conditions, redox-active metals such as iron and copper are strictly sequestered and occur at very low concentrations in their free form. However, under pathological conditions, such as acute or chronic inflammation, their free concentration may increase and induce the generation of hydroxyl radicals [121].

The activation of Nrf2 also induces the process of autophagy, a conserved lysosomal “self-digestion” pathway for degrading damaged proteins or organelles [121,122]. In this context, the breakdown of damaged mitochondria is particularly significant, as these can otherwise lead to increased ROS formation or even induce apoptosis in cells [123]. The activation of autophagy by polyphenols, such as epigallocatechin gallate, can therefore help counteract oxidative stress and the inflammation caused by it [124]. During autophagy, amino acids, fatty acids, and nucleotides are also released in lysosomes, which can then be used for protein synthesis and ATP production during stressful conditions [122].

The positive effects of polyphenols on inflammation are primarily mediated by the direct inhibition of NF-κB, the master regulator of inflammation [125]. This inhibition leads to the reduced production of pro-inflammatory cytokines (such as TNF-α or IL-6) and APPs (such as C-reactive protein) [125,126,127]. Under normal conditions, NF-κB is restricted to the cytoplasm, forming a complex with its inhibitor (IκBα). However, various stimuli can initiate its dissociation through inhibitors of kappa kinase β (IKKβ) and α (IKKα), which phosphorylate IκBα, leading to polyubiquitination and subsequent degradation. Once NF-κB is released, it becomes activated and translocates to the nucleus, where it binds to specific DNA regions known as NF-κB sites [128]. This binding is responsible for the expression of cytokines, adhesion molecules, and inflammatory enzymes [129]. Among the key stimuli triggering the NF-κB cascade are Toll-like receptors (TLRs), transmembrane proteins predominantly present in immune cells, which react to cytokines, ROS, and notably LPS [118]. Polyphenols interfere with various processes involved in NF-κB activation. On the one hand, they can inhibit the expression of NF-κB, and on the other, they also suppress its transactivation. The inhibition of transactivation is linked to the reduced expression of IKKβ, the kinase responsible for NF-κB release prior to its transport to the nucleus, as well as the suppression of TLR-4 signaling [118].

Anti-inflammatory effects of polyphenols are also indirectly triggered by Nrf2. The activation of Nrf2 not only activates antioxidant and cytoprotective pathways, as outlined above, but also induces strong anti-inflammatory effects [47,130]. Several target genes induced by Nrf2 are involved in both the suppression of pro-inflammatory pathways and the potentiation of anti-inflammatory pathways. Important inflammatory mediators and enzymes negatively regulated by Nrf2 are cytokines (such as IL-1, IL-6, and TNF-α), chemokines (such as CXC and CC), cell adhesion molecules (such as ICAM-1 and VCAM-1), matrix metalloproteinases, cyclooxygenase (COX)-2, and inducible nitric oxide synthase. Heme oxygenase-1 (HO-1) is a potent anti-inflammatory target, whose expression is upregulated by Nrf2 [45,131,132].

**Figure 2:**
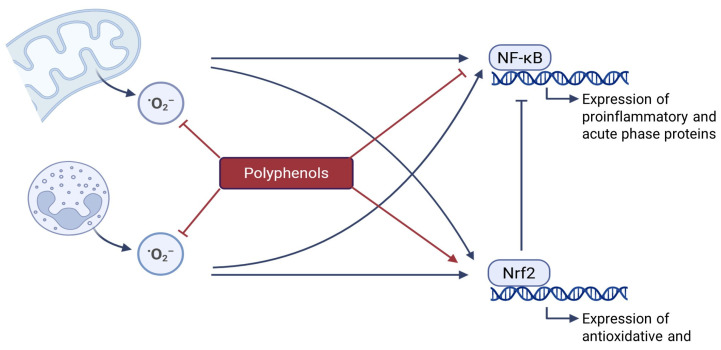
A simplified illustration of the effects of polyphenols in preventing oxidative stress and inflammation. Reactive oxygen species (ROS), such as superoxide radicals, are produced in mitochondria and by activated neutrophils, among other sources. These ROS can cause tissue damage and promote inflammation by activating NF-kB. However, polyphenols can directly neutralize ROS and activate Nrf2. Nrf2 not only induces antioxidant and cytoprotective pathways but also acts as an antagonist to NF-kB, enhancing anti-inflammatory effects. Created in BioRender. Eder, K. (2025) https://BioRender.com/wsmhdqf, accessed on 25 April 2025.

Since inflammation is also directly triggered by oxidative stress, the prevention of oxidative stress by polyphenols further protects against the onset of inflammation [95]. Additionally, polyphenols can activate transcription factors such as peroxisome proliferator-activated receptor γ, which inhibits inflammation by blocking the activation of NF-κB [133]. The role of polyphenols in the inhibition of oxidative stress and inflammation is shown in Figure 2.

## 3. Polyphenols in Grapes and Grape By-Products

Grapes are among the most widely cultivated fruits in the world. In 2023, global grape production amounted to 72.5 million tons, of which about 75% was used for wine production [134]. Grapes are one of the fruits with the highest content of natural polyphenols. Polyphenols are complex molecules representing a large group of over 8000 different components, all of which have a phenolic ring as a structural characteristic. According to the number of phenol rings and the structural elements binding these rings together, polyphenols can be classified as flavonoid-type and non-flavonoid-type polyphenols [135]. Flavonoids, which predominate in grapes, constitute the largest group of polyphenols, with over 4000 representatives. Flavonoids are plant pigments, but within the plant, they also exert antioxidant, antimicrobial, and light-screening functions. Their common property is two benzene rings connected by three carbon atoms, forming an oxygenated heterocycle. Depending on the chemical structure, oxidation degree, or unsaturation of the heterocyclic ring C, flavonoids can be classified into six subclasses: flavan-3-ols, flavonols, anthocyanes, flavones, isoflavons, and flavanones. These molecules are generally water-soluble, and they occur in glycosylated or aglycon form. Their basic structure is the flavone ring [56]. Examples of flavonoids, whose physiological effects have also been frequently studied, include flavonols such as quercetin and myricetin; flavones such as orientin, vitexin, and homoorientin; flavanols such as catechin, epicatechin, and epigallocatechin; the flavanone naringenin; the anthocyanin cyanidin; and the isoflavones genistein and daidzein [121,136,137]. Within the group of non-flavonoids, stilbenes and phenolic acids are the most important representatives [138]. Stilbenes are known as phytoalexins, protective compounds secreted by the plant following contact with a pathogen or abiotic stress [56]. Notable examples of stilbenes are resveratrol, trans-piceid, and trans-viniferins. Examples of phenolic acids, which also contribute to the defense of plants against pathogens such as bacteria, fungi, or viruses, are hydroxycinnamic and hydroxybenzoic acids [138].

Grape pomace, also known as grape marc or wine pomace, is an important by-product of the winemaking process, representing about 20–25% of the total grape weight used in wine production. This by-product consists of a mixture of grape skins (43% of total grape pomace), seeds (23%), stems (25%), and pulp remnants [9]. Globally, around 8.5 million tons of grape pomace are produced [139]. Grape pomace is rich in various nutrients, but the levels of individual nutrients can vary significantly depending on the maturity level, environmental factors, grape variety, and technology used in the winemaking process [16]. The main component of grape pomace is crude fiber, with a content of around 40% of dry matter on average. The contents of crude protein and crude fat are, on average, 12% and 8% of dry matter. Grape pomace is also rich in several minerals, such as potassium (20 g/kg dry matter), phosphorus (14 g/kg dry matter), and calcium (4 g/kg dry matter) [16]. The metabolizable energy (ME) content of grape pomace strongly depends on the fiber content, which leads to a decrease in the digestibility of organic matter. Since the fiber content varies over a wide range (14–75% of dry matter), the metabolizable energy content also fluctuates across a broad range. For pigs and poultry, an average ME content of 6.7 MJ per kg dry matter was reported (range: 5.1 to 8.7 MJ per kg dry matter) [16]. In a study in sheep, the metabolizable energy content of pomaces from red and white grapes was 5.5 and 6.1 MJ ME/kg dry matter, respectively [140]. Thus, overall, the ME content of grape pomace is low in both monogastric animals and ruminants.

Grape pomace can be divided into two fractions: seedless pomace (residual pulp, stems, and skin), accounting for 48–62% of total mass, and seeds, accounting for 38–52%. Grape seeds contain very high amounts of crude fiber (47% of the dry matter), most of which is indigestible, but they also contain fat (13% of the dry matter) with a high proportion of unsaturated fatty acids (especially linoleic acid) and protein (11% of the dry matter) [16]. The metabolizable energy content is even lower than that of grape pomace due to the low digestibility of the organic matter (4.7 to 6.9 MJ per kg dry matter in pigs and chickens) [16]. Grape seed extract and grape seed oil are two by-products derived from grape seeds. Grape seed extract is obtained when grape seeds from grape juice or wine processing are extracted, dried, and purified to produce a residue enriched in polyphenols [16].

During grape processing, existing polyphenols mainly remain in the grape pomace due to incomplete extraction. Therefore, grape pomace contains high levels of polyphenolic components. The total amount of polyphenols and the polyphenolic composition of different grape pomace types can vary greatly depending on the grape cultivar, soil type, weather, geographical location, and winemaking process [141,142]. Analyses of various red and white grape pomaces indicated that the total polyphenol content in grape pomace ranged between 15 and 80 mg per gram of dry matter [143,144,145,146]. The main representatives of polyphenolic compounds in this by-product are anthocyanins (only in red grape pomaces), catechins, flavonol glycosides, and phenolic acids [147]. The concentrations of individual polyphenolic compounds in different grape pomace samples can vary over a wide range, by a factor of 10 or even more [148]. Within the individual components of grape pomace, the highest levels of polyphenolic components are found in grape skins and seeds. Analyses of polyphenols in Italian red cultivars showed total flavonoid contents in seeds of around 100 to 160 mg per gram of dry matter, with proanthocyanidins being the main component. In skins, total flavonoid levels ranged from 30 to 50 mg per gram, with proanthocyanidins also being the main component [149]. In an analysis of 30 Chinese and Californian grape varieties, total polyphenol levels in skins ranged from 2 to 16 mg gallic acid equivalents per gram, while in seeds, the levels ranged from 30 to 57 mg gallic acid equivalents per gram. In this study, the antioxidant activities of grape skins and seeds of various varieties in vitro were strongly correlated with their contents of total polyphenols [150].

## 4. The Effects of Grape By-Products on Oxidative Stress and Inflammation in Farm Animals

In the following chapter, studies are presented that examine the effects of grape by-products on the antioxidant system and inflammation in pigs, chickens, and cattle. The studies mentioned are the result of a literature search in the databases PubMed and Google Scholar, using the search terms “grape” in combination with “pig”, “chicken”, and “cattle”. Among the multitude of results, studies were selected in which at least one parameter related to the antioxidant system or inflammation was considered.

### 4.1. Pigs

In recent years, a number of studies have been conducted with the aim of investigating the effects of grape by-products on the antioxidant defense system and their ability to reduce oxidative stress in pigs. Some of these studies also addressed the inflammation process. Different grape by-products were used in these investigations, including grape pomace, grape seeds, and grape seed extracts. In some studies, many parameters of the antioxidant system were examined, sometimes across multiple tissues, while in others, only individual parameters were investigated, and in some cases, in only a few tissues or in plasma alone. In some studies on fattening pigs, the antioxidant status of pork was also examined. An overview of studies investigating the effects of grape by-products on the antioxidant system and inflammation in pigs is given in Table 1.

#### 4.1.1. Studies in Weaned Pigs

Oxidative stress and inflammation in the intestine are particularly significant for piglets during the weaning phase. This phase is critical and stressful for piglets, often leading to enteric infections and gut disorders associated with inflammation and diarrhea [151,152]. Several studies have shown that grape by-products can mitigate oxidative stress and inflammatory processes in weaned piglets, particularly in the intestine, but also in other tissues.

In a study by Gessner et al. [153], administering grape seed and grape marc meal extract (1% in the diet) resulted in reduced NF-kB activity and a significant decrease in the expression of several pro-inflammatory genes in the duodenal mucosa. Interestingly, the authors also observed a reduction in Nrf2 activity and the expression of several Nrf2 target genes, attributed to lower inflammatory stress. Feed efficiency in pigs fed grape seed and grape marc meal extract was improved in this study. Similar results were found in a follow-up study by these authors, where the use of grape seed and grape marc meal extract (1% in the diet) led to the decreased expression of several pro-inflammatory genes (*TNF*, *IL8*, *IL1B*, *ICAM1*) in the mucosa in various intestinal sections (duodenum, ileum, colon) [154]. Feed efficiency showed a tendency for improvement when feed was supplemented with grape seed and grape marc meal extract (*p* < 0.10). Wei et al. [155] investigated 21-day-old weaned pigs fed diets supplemented with 50, 100, or 150 mg of grape seed procyanidins per kg, finding dose-dependent increases in SOD expression and activity in the jejunal mucosa, while the concentration of malondialdehyde (MDA), a marker of lipid peroxidation, decreased dose-dependently in this tissue. Although other antioxidant enzyme activities remained unchanged, the study suggested that grape seed procyanidins could reduce oxidative stress in the intestines of early-weaned piglets. Additionally, this study revealed a notable increase in microbiota diversity across various intestinal segments. No weight development data for the animals were reported in this study. In a parallel study by the same group with an identical experimental design, supplementation with grape seed procyanidins reduced diarrhea incidence, comparable to the effect of an antibiotic compound [156]. Furthermore, grape seed procyanidins improved antioxidant enzyme activities (SOD, GPx), total antioxidant capacity, and plasma concentrations of MDA, immunoglobulin (Ig) G, IgM, complement C4, and IL-2, which were interpreted by the authors as indicators of enhanced cellular and humoral immune responses. Despite these positive findings, performance data remained unchanged compared to control animals. Han et al. [157] demonstrated that supplementation with 250 mg of proanthocyanidins per kg diet in piglets, weaned after 28 days, increased glutathione (GSH) concentrations, increased SOD and GPx activities, and reduced MDA concentrations in the intestinal mucosa and serum. Proanthocyanidine feeding also increased intestinal microbiota diversity, strengthened gut barrier function, and improved performance (body weight gain increased, and the feed–gain ratio was reduced). Chedea et al. [158] reported that feeding a diet with 5% grape pomace to piglets weighing 10 kg enhanced CAT, SOD, and GPx activities, increased total antioxidant capacity, and reduced concentrations of thiobarbituric acid-reactive substances (TBARS), another indicator of lipid peroxidation, in the duodenum and colon. No differences were observed in performance data between the groups in this study. Rajkovic et al. [159] conducted an extensive study on the effects of grape extract (150 mg/kg diet) in weaned piglets. Positive effects on the villus surface in the ileum and jejunum were observed, but no effects were noted on gene expression, the activities of GPx and SOD, or TBARS concentrations in the liver, jejunum, and ileum at various time points (days 27/28, 55/56). Similarly, the expression of various tissue repair and immune response genes (*HSP70*, *HSP90AA1*, *CYP8B1*, *MMP13*, *TNFRSF14*, *CCL4*) in the liver, as well as antioxidant measures (SOD, MDA) and acute-phase proteins (haptoglobin, pigMAP) in plasma, remained unchanged by grape extract in the feed.

In addition to studies primarily investigating the effects of grape by-products on antioxidant and anti-inflammatory properties in the intestine, several investigations have also examined their effects in the serum or other organs of weaned pigs.

Kafantaris et al. [160] conducted an experiment with early-weaned piglets (4.8 kg body weight) fed diets supplemented with ensiled grape pomace. After feeding for 15 or 30 days, the treatment group showed a significant increase in GSH concentrations and a reduction in TBARS and protein carbonyl concentrations in various tissues (liver, heart, quadriceps, brain, spleen, kidneys, lungs, stomach, pancreas). However, antioxidant parameters in blood samples (activity of CAT; concentrations of GSH, TBARS, and protein carbonyls; total antioxidant capacity) remained largely unchanged. The feed conversion ratio improved in the first 15 days of feeding with grape pomace, but no longer improved after 30 days. Pistol et al. [161] investigated the effects of grape seed meal on the antioxidant status of 21-day-old pigs whose immune systems were challenged with dextran sulfate. In this study, grape seed meal increased the gene expression and activities of antioxidant enzymes (CAT, SOD, GPx) in the colon and lymph nodes while reducing DNA oxidative damage and protein carbonylation in these tissues. The authors attributed this antioxidant effect to Nrf2 activation in these tissues. Gessner et al. [162] studied the effects of grape seed and grape marc meal (1% in the diet) on a broad set of genes involved in inflammation, cytoprotection, and ER stress in the liver of piglets (10 kg body weight). Supplementation showed no effects on these metabolic pathways in the liver. The levels of NF-kB-p50, an active component of NF-kB, were unchanged, indicating no influence of grape seed and grape marc meal on inflammation in the liver. Antioxidant parameters in the liver and plasma (antioxidant capacity, α-tocopherol, and TBARS concentrations) also remained unaffected. In a study by Taranu et al. [163], a diet with 5% grape pomace was examined for its effects on liver parameters related to antioxidant status and inflammation in pigs (initial weight not specified). Grape pomace supplementation led to the reduced gene expression of *IL-8*, *IL-6*, *IFN-γ*, *eNOS*, and *COX2*, as well as lower protein concentrations of IL-8, TNF-α, and interferon (IFN)-γ. However, the activities of antioxidant enzymes (SOD, CAT, GPx) and total antioxidant capacity remained unchanged, while TBARS concentrations decreased. This study suggests that grape pomace exerts anti-inflammatory effects but has only moderate effects on the antioxidant system in the liver. Pistol et al. [164] studied piglets with an initial weight of 9 kg fed diets with 8% grape seed meal. This study revealed a marked reduction in the expression of several pro-inflammatory genes in the spleen. Antioxidant enzyme activities and total antioxidant capacity were also elevated, indicating both antioxidant and anti-inflammatory effects of grape seed meal in the spleen. In Park et al.’s study [165], peripheral blood mononuclear cells (PBMCs) from piglets on diets supplemented with grape seed-derived procyanidins released fewer pro-inflammatory cytokines (IL-1ß, IL-6, TNF-α) after an LPS challenge compared to PBMCs from control piglets. The authors interpreted these findings as an anti-inflammatory effect of procyanidins.

Taken together, the results of all the available studies suggest that grape by-products have a positive impact on the antioxidant system in weaned pigs and can reduce oxidative stress and inflammation, particularly in the intestine. Particularly strong effects of grape by-products on the antioxidant system and inflammation were observed in weaned piglets, in which weaning-associated inflammation processes in the intestine were enhanced. It is assumed that the strong effects, especially the reduction in inflammatory processes through grape by-products, are not only due to the antioxidant and anti-inflammatory effects of polyphenols but also due to an influence on the intestinal microbiome. Several studies have shown that grape by-products positively influence the composition of the microbiome in piglets, thereby suppressing pathogenic microorganisms [157,160,166,167].

Although the data are less consistent, grape by-products also showed beneficial effects on the antioxidant system in tissues other than the intestine. At least some of the studies also indicate that grape by-products can improve performance, particularly feed efficiency, in weaned pigs.

#### 4.1.2. Studies in Pigs with Body Weight Gains Greater than 30 kg and Growing-Finishing Pigs

In addition to studies performed on weaned pigs, there are also several studies dealing with the effects of grape by-products on the antioxidant system and inflammatory markers in pigs with weights greater than 30 kg and finishing pigs.

In a study by Zheng et al. [168], grape seed anthocyanidins at concentrations of 15, 30, 60, or 120 mg/kg diet were used in pigs with a body weight of 30 kg. The supplementation resulted in a linear increase in SOD and GPx activities in plasma and a reduction in MDA concentration. The feed-to-gain ratio was also improved, possibly due to the increased digestibility of energy and nutrients in the diet. Taranu et al. [169] provided pigs (76 kg body weight) with a diet containing 5% grape seed cake. This study showed that while SOD and GPx activities and total antioxidant capacity in the liver remained unchanged, pigs fed grape seed cake had reduced expression and protein concentrations of NF-kB and various cytokines (IL-1ß, IL-8, IL-6, TNF-α, IFN-γ) in the liver compared to the control group. The concentration of the phosphorylated, active NF-kB was also reduced, indicating anti-inflammatory effects in the liver due to grape seed cake supplementation. Horodincu et al. [170] investigated the effects of grape pomace (1, 5, 10, or 15 g/kg diet) on intestinal antioxidant and inflammatory status in growing–finishing pigs (around 85 kg body weight). This study showed dose-dependent reductions in NF-kB p65 expression and pro-inflammatory gene expression (*TNFa*, *IL-1ß*, *MHC-II*), along with the upregulation of Nrf2 in various intestinal segments (duodenum, jejunum, ileum, cecum). These findings suggest that grape by-products have beneficial effects on intestinal inflammatory status not only in weaned piglets but also in growing-finishing pigs.

Some studies have also explored the effects of grape by-products on pork quality, particularly regarding antioxidant status and susceptibility to lipid peroxidation. Yan and Kim [171] fed pigs (20 kg body weight) diets supplemented with fermented grape pomace products (30 g/kg) for 15 weeks, reaching a final weight of 106 kg. The supplementation significantly improved daily weight gain during the grower phase and tended to improve feed efficiency compared to controls. Pork (*M. longissimus dorsi*) showed no differences in marbling and firmness, but TBARS concentrations were significantly lower in pigs whose diets were supplemented with grape by-products. Trombetta et al. [172] investigated the effects of supplementation of ensiled grape pomace (3.5 or 7%) in pigs, whose diets were enriched with 3% linseed oil as a prooxidative challenge. The nutrient contents (lipids, protein, cholesterol, ash), texture, and color of pork were unaffected by supplementation. The concentration of TBARS in pork was unexpectedly elevated following grape pomace supplementation, possibly as a result of increased levels of polyunsaturated fatty acids. Tian et al. [173] fed pigs (55 kg body weight) diets supplemented with 6% dried grape pomace for 75 days, reaching a final weight of 115 kg. Dried grape pomace feeding increased total antioxidant capacity and SOD activity and reduced MDA and ROS concentrations in pork (*M. longissimus thoracis*) compared to the control group.

Although there are significantly fewer studies on heavier pigs compared to weaned pigs, overall, these studies suggest that grape by-products may have beneficial effects on the antioxidant system and inflammation. Additionally, the available studies indicate that the use of grape by-products in growing-finishing pigs can slow down the formation of lipid peroxidation products in pork during storage. Although the concentrations of polyphenols in pork were not measured in the cited studies, it seems plausible that the lower susceptibility to oxidation is due to an accumulation of polyphenols in the muscle. Polyphenols can then scavenge ROS that arise during oxidation in pork during storage and slow down the heme-mediated lipid oxidation process [12,174,175].

**Table 1:** Overview of studies dealing with the effects of grape by-products on the antioxidant system and inflammation in pigs.

Species	Grape By-Product	Dose and Treatment Duration	Main Effects	Reference
Weaned pigs	Grape pomace	5% in diet for 36 days	Duodenum: ↑ SOD activity Colon: ↑ CAT and GPx activities ↑ Total antioxidant status, ↓ TBARS	[158]
Weaned pigs	Ensiled grape pomace	Unspecified dose for 15 or 30 days	d1-d15: ↑ ADG, FCR d1-d30: ↑ GSH, TBARS, and protein carbonyls in different tissues	[160]
Weaned pigs	Grape seed meal	8% for 30 days	Colon and lymph nodes: ↑ CAT, SOD, GPx gene expression and/or activity ↓ DNA oxidative damage and protein carbonylation	[161]
Weaned pigs	Grape seed and marc meal extract	1% in diet for 28 days	Intestinal mucosa: ↓ NF-κB and Nrf2 transactivation, ↓ NF-κB and Nrf2 target gene expression, ↑ FCR	[157]
Weaned pigs	Grape seed and marc meal extract	1% in diet for 28 days	↓ Pro-inflammatory gene expression (*TNF*, *IL8*, *IL1B*, *ICAM1*) in the intestinal mucosa	[154]
Weaned pigs	Grape seed and marc meal extract	1% in diet for 28 days	Liver and plasma: No effect on TEAC, α-tocopherol, TBARS Liver: No effect on expression of genes involved in inflammation, cytoprotection, and ER stress, and NF-kB-p50 protein level	[162]
Weaned pigs	Grape extract	150 mg/kg diet for 27/28 or 55/56 days	No effects on GPx and SOD activity and TBARS in liver, jejunum, and ileum No effects on tissue repair or immune response-related gene expression in liver (*HSP70*, *HSP90AA1*, *CYP8B1*, *MMP13*, *TNFRSF14*, *CCL4*) No effect on SOD and MDA level and acute-phase proteins in plasma	[159]
Weaned pigs	Grape seed procyanidins	50, 100, or 150 mg/kg diet for 28 days	↓ MDA; ↑ GPx; ↑ SOD ↑ Microbiota diversity ↓ Diarrhea incidence ↑ Serum total antioxidant capacity	[155,156]
Weaned pigs	Grape seed-derived procyanidins	100, 200, or 400 mg/kg diet for 56 days	↓ IL-1β, IL-6, TNF-α levels in PBMCs after LPS challenge	[165]
Weaned pigs	Proanthocyanidins	250 mg/kg for 28 days	↑ GSH, SOD, GPx, and ↓ MDA in intestinal mucosa and serum ↑ ADG, FCR	[157]
Growing pigs	Grape seed anthocyanidins	15, 30, 60, 120 mg/kg for 33 days	↑ Plasma SOD, GPx, ↓ MDA ↑ ADG ↑ FCR (30–120 mg/kg groups)	[168]
Finishing pigs	Grape pomace	1, 5, 10, or 15 g/kg diet for 90 days	↓ Intestinal NF-κB p65 and pro-inflammatory target gene expression ↑ Intestinal Nrf2 expression ↑ ADG, ADFI	[170]
Finishing pigs	Grape seed cake	5% in diet for 24 days	Liver: ↓ Expression of cytokines (*IL-1ß*, *IL-8*, *IL-6*, *TNF-α*, *IFN-γ*) and NF-κB and target genes ↓ CAT expression and activity No effect on SOD and GPx activities and total antioxidant capacity	[169]
Finishing pigs	Grape pomace	5% in diet for 24 days	Liver: ↓ Expression of cytokines and NF-κB target genes; (*IL-8*, *IL-6*, *IFN-γ*, *eNOS*, *and COX2*) ↓ Protein concentrations of IL-8, TNF-α, and interferon (IFN)-γ; No effect on SOD, CAT, GPx, and total antioxidant capacity; ↓ TBARS	[163]
Finishing pigs	Dried grape pomace powder	6% in diet for 75 days	↑ Total antioxidant capacity, SOD ↓ MDA and ROS in pork	[173]
Finishing pigs	Fermented grape pomace	30 g/kg for 105 days	↓ TBARS in pork ↑ ADG during grower phase	[171]
Sows	Grape seed polyphenols	200 or 300 mg/kg for 56 days	↑ SOD, GPx in plasma; ↑ IgG and IgM in colostrum; ↑ Farrowing and pre-weaning piglet survivability	[176]

ADFI: average daily feed intake; ADG: average daily gain; CCL4: C-C motif chemokine ligand 4; CYP8B1: cytochrome P450 family 8 subfamily B member 1; eNOS: nitric oxide synthase 3; FCR: feed conversion ratio; HSP: heat shock protein; IFN-γ: interferon gamma; MMP13: matrix metallopeptidase 13; TNFRSF14: TNF receptor superfamily member 14; ↑ increase; ↓ decrease.

#### 4.1.3. Studies in Sows

Our literature search identified only one study that investigated the effects of grape by-products in sows with respect to the antioxidant system or inflammation. Wang et al. [176] provided sows with diets supplemented with 200 or 300 mg of grape seed polyphenols per kg during the period from day 80 of gestation to piglet weaning (day 21 of lactation). Supplementation led to increased SOD and GPx activities in plasma on day 110 of gestation; however, total antioxidant capacity and MDA concentrations remained unchanged. No inflammatory parameters were measured in this study. However, grape seed polyphenols increased IgM and IgG concentrations in the colostrum and pre-weaning piglet survivability.

### 4.2. Chickens

There are a number of studies on the effects of grape by-products on oxidative stress and inflammation in chickens (broilers and laying hens). Compared to the studies on pigs, most of the investigations, however, focused mainly on the antioxidant defense system, while few studies examined the impact of grape by-products on inflammation. An overview of these studies is presented in Table 2.

#### 4.2.1. Studies in Broilers

The literature contains several studies that deal with the effects of grape by-products on the antioxidant status in broilers. However, in the majority of these studies, only a few antioxidant parameters were considered, often limited to a few tissues, such as serum. Most of these studies used either grape seeds or polyphenols from grape seeds, such as procyanidins. These studies are described first, followed by other studies that used different forms of grape by-products.

Wang et al. [177] conducted a study in which broilers were fed diets supplemented with 100 mg of grape seed extract per kg. To induce oxidative stress, 3% oxidized rice bran oil was also added to the diets. The addition of grape seed extract resulted in an increase in GPx activity and total antioxidant capacity, as well as a reduction in MDA concentration in serum and the liver. However, the activities of CAT and SOD remained unchanged. Furthermore, in the group with added grape seed extract, the gene expression levels of *Nrf2* and its target genes (*HO-1*, *CAT*) in the liver were elevated. The effects of grape seed extract were weaker than those of vitamin E (25 mg/kg), which was added to the diet in a positive control group. The addition of grape seed extract improved performance parameters (body weight gain, average daily feed intake, feed-to-gain ratio) to a degree comparable to the positive control. Noor et al. [178] tested diets with 1, 2, or 3% grape seed powder in their study on broilers. They observed an increase in GPx activity and a decrease in MDA concentration in plasma, along with a rise in the average daily body weight gain of broilers. Gungor et al. [179] examined the effects of raw or fermented grape seed (5 g/kg diet) on antioxidant parameters in broilers. Raw grape seed led to an increase in GPx and CAT activities (but not SOD), while fermented grape seed did not. Both raw and fermented grape seeds improved the average daily body weight gain in comparison to the control group. Cao et al. [180] used two concentrations of a grape seed proanthocyanidin extract (GSPE, 200 and 400 mg/kg diet) in broilers. GSPE supplementation (at both concentrations) increased the activities of SOD and GPx, as well as the total antioxidant capacity in serum, while reducing MDA concentration. Supplementation with GSPE also reduced IL-1β concentrations in the serum, ileum, and jejunum. Additionally, it improved the average daily body weight gain, daily feed intake, and feed-to-gain ratio. Rajput et al. [181] studied the effects of GSPE (250 mg/kg diet) on the antioxidant system of broilers treated with aflatoxin B1 (1 mg/kg diet). Supplementation with GSPE increased the activities of SOD, GPx, CAT, GR, and glutathione S-transferase (GST), as well as GSH levels, while reducing MDA concentrations in the liver and serum. The same group investigated the effects of GSPE on inflammation in the same experimental model [182]. They observed that GSPE supplementation led to a significant reduction in pro-inflammatory cytokine expression (*TNF-α*, *IFN-γ*, *IL-1ß*, *IL-6*) in the spleen, an effect mediated by NF-kB inhibition. In the liver, an increase in the expression and concentrations of Nrf2 and some of its target genes (*HO-1*, *GPx1*, *NQO1*, *GCLC*) was observed, indicating that GSPE activated the antioxidant and cytoprotective systems through Nrf2 activation during an aflatoxin B1 challenge. In the animals challenged with aflatoxin B1, GSPE also significantly improved the average daily body weight gain, daily feed intake, and feed conversion ratio. In a control group not treated with aflatoxin B1, GSPE showed no effects on the antioxidant system or inflammation; however, the average daily gain was increased. Abu Hafsa and Ibrahim [183] tested polyphenol-rich grape seeds (10, 20, 40 g/kg diet) on the antioxidant system in broilers. They observed a dose-dependent increase in the activities of SOD, CAT, GPx, and GST, as well as an increase in GSH levels and a reduction in TBARS concentration in serum. Performance data (body weight gain, feed conversion ratio) were improved at concentrations of 10 and 20 g of polyphenol-rich grape seeds per kg compared to the control group, but were reduced at 40 g/kg. Farahat et al. [184] conducted a study on broilers using diets with grape seed extract concentrations ranging from 125 to 2000 mg/kg. Even the lowest supplementation level led to an increase in reduced GSH levels in the liver and a reduction in MDA concentration in meat. Higher doses did not provide additional benefits compared to 125 mg/kg. Wang et al. [185] studied the effect of a grape seed proanthocyanidin extract at a dose of 12 mg/kg diet on the antioxidant system of broilers infected with *Eimeria tenella*. They found that SOD activity in plasma was increased by the treatment, but MDA concentration remained unchanged. The daily weight gains of infected animals were significantly improved by the grape seed proanthocyanidin extract supplementation compared to the control. In a study by Yang et al. [186], the effects of grape proanthocyanidins (5.5 to 30 mg/kg diet) on the antioxidant system were investigated. In this study, only SOD activity and MDA concentration were measured. SOD activity in plasma was increased, even at the lowest dose. MDA concentration in plasma was reduced at 7.5 mg/kg while remaining unchanged at 15 and 30 mg/kg compared to the control.

In addition to studies where grape seeds or grape seed extracts were used as polyphenol sources, there are further investigations that utilized other grape by-products, such as grape pomace or grape extracts.

In a study by Mavrommatis et al. [187], ground grape pomace (25 g/kg diet), dried wine lees extract (2 g/kg diet), and extract from grape stems (1 g/kg diet) were included in the rations of broilers. The expression of antioxidant enzymes (*CAT*, *GPx*, *SOD*, *GST*) in the liver and their activities in plasma were not significantly influenced by the addition of these three different grape by-products. However, the concentration of MDA in breast muscle was reduced by the inclusion of dried wine lees extract or extract from grape stems. In a follow-up study by the same authors, the expression of genes related to inflammation (*NFKB*, *MAPK*, *TNF*, *TLR4*, *INFA*, *INFG*, *IL1B*, *IL2*, *IL8*, *IL18*) in the liver, bursa of Fabricius, and spleen was measured using the same experimental model [188]. Overall, all three grape by-products had little effect on the expression of these genes in the three tissues. Duangnumsawang et al. [189,190] used a grape extract as an additive in broiler diets. The content of the product was adjusted so that the diet contained 165 mg of procyanidins and 585 mg of total polyphenols per kg. This addition had no impact on the expression of various pro-inflammatory genes in the cecum and ileum. In a study by Brenes et al. [191], broilers were fed diets supplemented with different amounts of pomace grape extract (15, 30, 60 g/kg). The addition of pomace grape extract increased antioxidant capacity in the ileum but not in the serum, and it did not affect animal performance. Makri et al. [192] investigated the effect of grape pomace (used as a component of corn silage) on the antioxidant system in broilers. After 50 days of feeding grape pomace, an increase in GSH concentration was observed in the erythrocytes, heart, liver, lung, kidney, and spleen, along with an increase in total antioxidant capacity in the liver and kidney and a reduction in TBARS concentrations in the plasma, heart, quadriceps, intestine, and spleen compared to the control group. Gungor et al. [193] studied the effects of raw and fermented grape pomace (each 15 g/kg diet) in broilers. They observed an increase in GPx and SOD activities in serum after feeding raw grape pomace and an increase in CAT activity after feeding fermented grape pomace. Fermented grape pomace also led to an increase in the final weight of the animals, although the feed conversion ratio remained unchanged compared to the control group. In the work by de-Cara et al. [194], broilers were fed a supplement comprising olive tree leaves and grape by-products (2 g/kg diet). This preparation had only weak effects on the antioxidant system. The activity of SOD in plasma was increased, but the activities of CAT and GPx as well as the concentration of MDA remained unchanged, with no effects observed on performance parameters.

Several studies also investigated the impact of different grape by-products (grape pomace, grape seeds, grape skins) on the concentrations of lipid peroxidation products (TBARS, MDA) in broiler meat (thigh muscle, breast muscle). In most studies in which meat was frozen immediately after slaughter and thawed directly for analysis, broilers on diets supplemented with grape by-products showed no difference in lipid peroxidation product concentrations in meat [191,195,196,197]. Only in Turcu et al.’s study [198] was a lower concentration of TBARS observed in freshly thawed meat from broilers on diets supplemented with grape pomace compared to control animals. In meat stored in a refrigerator for 3 to 10 days, a significant reduction in MDA or TBARS concentration was observed due to grape product supplementation in several studies [191,195,196,197,199,200]. Feeding a preparation consisting of olive leaves and a grape-based product also reduced MDA concentrations in broiler meat after 6 days of storage at 4 °C [194]. These findings suggest that the deposition of polyphenols in meat following the feeding of grape by-products leads to the reduced oxidative sensitivity of lipids in the meat.

In sum, many studies have explored the effects of grape by-products on the antioxidant system and inflammation in broilers. Although the studies used different products with widely varying concentrations in the feed, the overall picture indicates that grape by-products also have beneficial effects on the antioxidant system in broilers and can reduce inflammation. Similar to pigs, broilers given grape by-products also showed positive effects on performance (body weight gain, feed efficiency) and favorable effects on the susceptibility of lipids to oxidation in meat in some studies.

#### 4.2.2. Studies in Laying Hens

Beyond the multitude of experiments conducted on broilers, there are some studies on the effects of grape by-products in laying hens. Most studies investigated whether grape by-products could influence egg-laying performance and egg quality. In some studies, reference was also made to the antioxidant system, though only a few relevant parameters were typically measured.

In Tufarelli et al.’s study [201], the effects of grape pomace (5% in the diet) on antioxidant parameters in laying hens were studied. The activities of GPx and SOD, total antioxidant capacity, and MDA concentration were unchanged between the treated and control groups. This study showed a slight but significant reduction in feed intake and egg mass due to grape pomace supplementation. Selim et al. [202] also studied the use of grape pomace (3, 6, and 9% in the diet) in laying hens. They observed a linear increase in GPx activity and a linear reduction in MDA concentration in both serum and egg yolk with increasing grape pomace content in the diet. This study also reported a linear increase in egg production, egg weight, egg mass, and feed intake, along with a linear decrease in feed conversion. Reis et al. [203] used grape pomace flour (1, 2, 3% in the diet) as a supplement in laying hens under heat stress conditions. Supplementation reduced TBARS concentrations in egg yolks and increased total antioxidant capacity dose-dependently. The activities of GPx and SOD in serum increased with supplementation, while the TBARS concentration decreased. In this study, egg-laying performance improved with 1% grape pomace flour supplementation but was unchanged with 2 or 3% compared to the control group. Herranz et al. [204] studied the effects of grape pomace (50 g/kg diet) in laying hens and observed no influence on α- and γ-tocopherol concentrations or polyphenol concentration (measured as gallic acid concentration) in egg yolk.

Some studies considered only the MDA or TBARS concentration in serum or egg yolk. In the studies by Hafeez et al. [205,206], grape seed extract supplementation (250, 500, 750 mg/kg diet) led to reduced MDA concentration in plasma. Kaya et al. [207] observed a reduction in MDA concentration in eggs stored for 14 days with the addition of grape seed (0.5, 1.0, 1.5% in the diet) and grape seed extract (675, 1350, 2025 mg/kg diet) in laying hens. Shorter (0 d, 7 d) or longer (28 d) storage periods showed no such effect. In a study by Kara et al. [208], supplementing the diets of hens with 4 or 6% grape pomace lowered the MDA concentration in the yolks of eggs stored for 15 d, but not in fresh eggs. Romero et al. [209] observed a reduction in TBARS concentration in four-month-stored eggs of laying hens fed diets supplemented with 60 g/kg of grape pomace but not in those of hens fed diets supplemented with 0.5 or 1.0 g/kg of grape extract. Grigorova et al. [210] observed no influence on MDA content in fresh eggs or eggs stored for 30 days in the refrigerator, but they found reduced MDA content in eggs stored at room temperature when hens were fed diets supplemented with grape marc flour (1 or 3% in the diet).

The number of studies regarding the effects of grape by-products on the antioxidant system in laying hens is significantly lower compared to broilers. The data on the effects on the antioxidant system and performance are also less consistent. However, there are several indications that the feeding of grape by-products inhibits the formation of lipid peroxidation products in egg yolk during storage.

### 4.3. Cattle

Compared to pigs and chickens, there are relatively few studies on the use of grape by-products in cattle feeding. The majority of these studies have focused on the effects of grape by-products on methane production [211,212,213] or on the quality of milk or its derived dairy products [214,215,216,217,218] in dairy cows. An overview of studies performed in cattle dealing with the antioxidant system and inflammation is shown in Table 3.

**Table 2:** Overview of studies dealing with the effects of grape by-products on the antioxidant system and inflammation in chickens.

Species	Grape By-Product	Dose and Treatment Duration	Main Effects	Reference
Broiler chickens	Grape pomace	15, 30, or 60 g/kg for 21 days	↑ Antioxidant capacity of ileal content No effect on ADG, ADFI, and FCR	[191]
Broiler chickens	Grape seed powder	1, 2, or 3% in diet for 42 days	2% and 3% in diet: ↑ Plasma GPx; ↓ Plasma MDA; ↑ ADG	[178]
Broiler chickens	Grape seed extract	100 mg/kg for 42 days	Serum and liver: ↑ GPx and total antioxidant capacity, ↓ MDA; Liver: ↑ Nrf2 target genes ↑ ADG, ADFI, FCR	[177]
Broiler chickens	Grape seed extract after *E. tenella* challenge	12 mg/kg for 21 days	Plasma: ↑ SOD activity, No effect on MDA conc. ↑ ADG	[185]
Broiler chickens	Grape seed proanthocyanidins	200 or 400 mg/kg for 21 days	Serum: ↑ SOD and GPx, ↓ MDA Serum, Ileum und Jejunum mucosa: ↓ IL-1β ↑ ADG, ADFI, FCR	[180]
Broiler chickens	Grape seed proanthocyanidins after aflatoxin B1 challenge	250 mg/kg for 28 days	Liver and serum: ↑ SOD, GPx, CAT, GR, GST, and GSH level ↓ MDA Spleen:↓ Inflammatory cytokines	[181]
Broiler chickens	Grape seed proanthocyanidins after aflatoxin B1 challenge	250 mg/kg or 500 mg/kg for 28 days	Spleen: ↓ Cytokine expression (*TNF-α*, *IFN-γ*, *IL-1β*, *IL-6*) Liver: ↑ Expression of Nrf2 and some target genes (*HO-1*, *GPx1*, *NQO1*, *GCLC*); ↑ ADG, ADFI, FCR	[182]
Laying hens	Grape pomace	3, 6, and 9% in diet for 8 weeks	↑ Feed efficiency; ↑ Egg mass; ↑ Egg weight 6% and 9% in diet: ↑ Feed intake ↑ Egg production; ↑ GPx and ↓ MDA in serum and egg yolk	[202]
Laying hens	Grape pomace	5% in diet for 4 weeks	No significant changes in egg tocopherol or polyphenol content	[204]
Laying hens	Grape pomace	5% in diet for 12 weeks	↓ Feed intake ↓ Egg mass No effect on serum GPx, SOD, MDA, and total antioxidant capacity	[201]
Laying hens	Grape pomace	4% or 6% in diet for 12 weeks	↓ MDA in plasma and yolk of eggs stored for 15 days	[208]
Laying hens	Grape pomace/Grape extract	30 or 60 g/kg/0.5 or 1 g/kg for 4 weeks	60 g/kg grape pomace: ↓ TBARS in eggs stored 4 months	[209]
Laying hens	Grape pomace flour under heat stress	1%, 2%, or 3% for 35 days	↓ TBARS in egg yolk (all inclusion level) and serum (2% and 3%); ↑ Total antioxidant capacity in egg yolk (all inclusion level) and serum (2% and 3%) ↑ Serum levels of GPx (all inclusion level) and SOD (2%) ↑ Egg-laying performance (1% inclusion level) ↑ Feed intake	[203]
Laying hens	Grape marc flour	1% or 3% in diet for 34 days	↓ MDA in eggs stored 30 days at room temperature No effect on MDA conc. in eggs stored 30 days under refrigeration	[210]
Laying hens	Grape seed extract	250, 500, or 750 mg/kg for 5 weeks	↓ MDA in plasma	[206]
Laying hens	Grape seed (GS) Grape seed extract (GSE)	0.5%, 1%, or 1.5%/675, 1350, or 2025 mg/kg for 12 weeks	GS 1%, GSE 1350 mg and 2025 mg/kg: ↓ MDA in eggs stored 14 days; GS 1.5%, GSE 2025 mg/kg: ↑ MDA in eggs stored 7 days	[203]

Abbreviations: see Table 1; ↑ increase; ↓ decrease.

#### 4.3.1. Studies in Dairy Cows

For high-yielding dairy cows, the transition period (from pregnancy to lactation) poses a significant metabolic challenge. Feed intake decreases even before calving, and especially at the onset of lactation, cows experience a severe negative energy balance, accompanied by sharply increased plasma concentrations of free fatty acids and ketone bodies, as well as elevated levels of triglycerides in the liver [219,220,221]. In addition to this metabolic stress, the liver of early lactating cows is exposed to various inflammatory challenges, such as microbial components, pro-inflammatory cytokines, and ROS. These inflammatory challenges arise from infectious diseases such as mastitis and endometritis, as well as conditions such as subacute rumen acidosis and abomasal displacement, which frequently occur during parturition, the onset of lactation, or both [59,222,223,224]. Consequently, transition dairy cows develop inflammation-like conditions in the liver, characterized by the induction of an acute-phase response [57,59,223,225,226]. These pro-inflammatory conditions also lead to oxidative stress and ER stress in the liver [21,114,227,228]. Pro-inflammatory conditions, in combination with oxidative and ER stress, affect not only the health but also the performance of high-yielding dairy cows [59].

Several studies have investigated the impact of grape by-products on oxidative stress and inflammatory processes in dairy cows. In Gessner et al.’s study [229], the use of grape seed and grape marc meal extract (1% in the total mixed ration) was extensively analyzed for its effects on the antioxidant and inflammatory status and additionally on the occurrence of ER stress in the liver of dairy cows 1 and 3 weeks postpartum. Numerical reductions in the expression of the UPR (as an indicator of ER stress) and inflammatory genes were observed in the liver of cows fed the supplement, but they were not significant. The plasma concentrations of various antioxidants and TBARS and the total antioxidant capacity were also not significantly affected by grape seed and grape marc meal extract supplementation. However, the expression of FGF21, a stress hormone [102,230], was reduced at weeks 1 and 3 postpartum, indicating that metabolic stress was mitigated by grape seed and grape marc meal extract supplementation. In another study by the same group, a transcriptional analysis of liver samples was conducted one week postpartum in cows on a diet supplemented with grape seed and grape marc meal extract (1% in the total mixed ration) [231]. Among the most downregulated genes were those associated with the UPR and inflammation in the liver of cows given grape seed and grape marc meal extract. The plasma concentrations of serum amyloid A and haptoglobin, two acute-phase proteins, were also reduced. The authors concluded that the grape product could counteract the development of inflammation and ER stress in the liver during early lactation. Gorbert et al. [232] provided cows with diets containing linseed oil and vitamin E, along with a plant extract rich in polyphenols. This extract included grape components, among other ingredients (rosemary, citrus, marigold). Supplementation lowered the susceptibility of plasma lipids to oxidation (lag phase) and reduced MDA levels in plasma. However, it is unclear to what extent these effects were due to the grape extract, as the preparation also contained other polyphenol sources. Signor et al. [233] supplemented Jersey heifers daily with 25 mL of grape seed oil (compared to soybean oil in the control group). Grape seed oil supplementation resulted in lower TBARS concentrations and higher antioxidant capacity in plasma postpartum, with no impact on feed intake or milk production. Chedea et al. [214] investigated the effects of grape pomace (15% in the diet) on general health status and milk quality. They observed increased plasma polyphenol levels in cows on a diet supplemented with grape pomace. Other parameters of the antioxidant system or inflammation-related parameters were not measured. In Huang et al.’s study [234], cows were fed diets supplemented with GSPE (20, 40, 60, 80 mg/kg body weight per day). Feed intake was unaffected by supplementation with GSPE, as were the activities of antioxidant enzymes (GPx, SOD), total antioxidant capacity, and plasma MDA concentration. However, the milk yield was increased by supplementation with 20 mg GSPE/kg body weight per day.

It should be noted that the number of studies on the effects of grape by-products in dairy cows is, overall, quite limited. There are some indications that grape by-products can strengthen the antioxidant system in cows and counteract ER stress in the liver. However, the available data are insufficient to make a clear statement on this.

#### 4.3.2. Studies in Calves and Beef Cattle

In addition to studies in dairy cows, there are a few investigations dealing with the effects of grape by-products on the antioxidant system in calves or beef cattle. Urkmez and Biricik [235] supplemented 3-day-old heat-stressed female calves with grape seed extract (25, 50, 100 mg/kg body weight/day). They observed a dose-dependent reduction in plasma MDA and TNF-α concentrations and an increase in plasma SOD activity. Other inflammatory and antioxidant plasma parameters showed no response to grape seed extract supplementation. The authors concluded that supplementation with grape seed extract improves the antioxidant and inflammatory status in calves. Ma et al. [236] provided beef calves (68 days old) with 4 g of grape seed extract daily. Grape seed extract supplementation had no impact on feed intake or weight gain but significantly affected average daily gain, microbial protein production in the rumen, and dry matter digestibility. After 60 days of feeding, the activities of antioxidant enzymes (CAT, SOD) and total antioxidant capacity in plasma were increased, while MDA concentrations were reduced. IgG and IgA levels were elevated, and cytokine concentrations (TNFα and IL-6 significantly; IL-1β and IL-10 in trends) were reduced. The authors concluded that grape seed extract enhances antioxidant capacity and immunity in beef cattle. Iannaccone et al. [237] provided 4-month-old Friesian cattle with a diet supplemented with 10% grape pomace flour. A whole-transcriptome analysis of blood samples revealed the significant upregulation of IL-1 and NF-kB signaling, indicating immune system activation. However, pro-inflammatory cytokine concentrations were not measured, so this indication remains speculative. Additionally, MDA concentrations in meat, particularly after 7 days of storage at 4 °C, were significantly reduced, suggesting inhibited lipid peroxidation of meat lipids during storage. Molosse et al. [238] fed 9-month-old steers diets containing 10% grape pomace bran (GPB) or grape pomace silage (GPS) and examined antioxidant parameters in the serum, intestine, and liver. The results were inconsistent: some parameters (TBARS in serum and liver, GST and ROS in liver) improved with GPS, while others (GST in serum, intestine, and liver) improved with GPB. Performance (weight gain, feed conversion ratio) was worsened by GPB but unaffected by GPS. Li et al. [239] fed 16-month-old Angus bulls (with a weight of around 580 kg) a diet supplemented with 100 or 200 g of dried grape pomace per kg of total mixed ration for a period of 141 d. In general, they observed no beneficial effects of the supplementation on the antioxidant system or the concentration of ROS or MDA in serum. Moreover, supplementation with 200 g of dried pomace per kg of total mixed ration actually lowered daily body weight gains and increased the food-to-gain ratio.

Similar to studies in dairy cows, the number of studies in calves and beef cattle on the effects of polyphenols on the antioxidant system and inflammation is low overall, and the effects are also inconsistent.

**Table 3:** Overview of studies dealing with the effects of grape by-products on the antioxidant system and inflammation in cattle.

Species	Grape By-Product	Dose and Treatment Duration	Main Effects	Reference
Dairy cows	Grape pomace	15% for 12 weeks	↑ Plasma polyphenol conc. No effect on milk polyphenol conc.	[214]
Dairy cows	Grape seed and marc meal extract	1% in total mixed ration for 12 weeks	Plasma: No effects on concentrations of various antioxidants, TBARS, and total antioxidant capacity Liver: ↓ FGF21 expression	[229]
Dairy cows	Grape seed and marc meal extract	1% in total mixed ration for 4 weeks	↓ Plasma acute-phase proteins (SAA, HP) ↓ Expression of hepatic genes related to inflammation and ER stress	[231]
Dairy cows	Grape seed extract (dissolved in drinking water, 500 mL per os)	20, 40, 60, or 80 mg/kg BW/day for 50 days	No effects on GPx, SOD, total antioxidant capacity in serum, and MDA in plasma; no effects on ADFI; ↑ Milk yield (20 mg/kg/BW/day group)	[234]
Calves	Grape seed extract	25, 50, and 100 mg/kg BW/day for 60 days	↓ Plasma MDA and TNF-α; ↑ Plasma SOD	[235]
Calves	Grape seed extract	4 g/day for 60 days	↑ Plasma SOD, CAT, total antioxidant capacity, IgG and IgA; ↓ Plasma MDA, TNF-α, IL-6; ↑ ADG	[236]
Beef cattle	Grape pomace flour	10% in diet for 75 days	↓ MDA in meat after 7 d storage; ↑ IL-1 and NF-κB signaling	[237]
Beef cattle	Grape pomace bran (GPB) or grape pomace silage (GPS)	10% in diet for 21 days	GPB: ↓ TBARS in serum, TBARS in liver, GST and ROS in liver; ↓ ADG; ↑ FCR; GPS: ↓ GST in serum, intestine, liver	[238]
Beef cattle	Dried grape pomace	100 or 200 g per kg TMR for 129 days	200 g group: ↓ ADG; ↑ FCR	[239]

See abbreviation Table 1; HP: haptoglobin; SAA: serum Amyloid A; ↑ increase; ↓ decrease.

## 5. Conclusions and Future Perspectives

It is well known that oxidative stress and inflammation, resulting from high-performance, intensive animal husbandry, and additional stress factors, can significantly affect not only the productivity but also the health of farm animals [240,241,242]. Numerous studies presented in this review show that grape-derived products, which are primarily by-products of winemaking, have the potential to counteract oxidative stress and inflammation in farm animals. Although grape-derived products have a complex nutritional composition, it is assumed that their beneficial effects on oxidative stress and inflammation are primarily due to their high polyphenol content. As described in the section “Effects of polyphenols on oxidative stress and inflammation,” the antioxidant and anti-inflammatory effects of polyphenols and their biochemical basis are well documented.

Most polyphenols generally exhibit low absorption rates in the small intestine. Each class of polyphenols has its own unique chemical structure that results in specific solubility and lipophilicity, which in turn affects its bioavailability. However, it has been estimated that less than 5% to 10% of plant polyphenols are absorbed in the small intestine [243,244]. Additionally, polyphenols that have been absorbed are recognized as xenobiotic compounds. A proportion of the absorbed polyphenols are directly subjected to biotransformation in enterocytes. Upon passing through the portal vein, the remainder is largely subjected to glucuronidation, methylation, or sulfation in the liver as part of the natural detoxification process for xenobiotics [245,246]. These conjugated polyphenol metabolites are then transported back to the gastrointestinal tract through the bile duct to be further metabolized and/or excreted as feces [246]. Several other tissues (including lung, kidney, and brain) are also capable of polyphenol biotransformation [245]. Therefore, the concentrations of polyphenols in plasma and tissues are relatively low. Because polyphenol concentrations are higher in intestinal cells, the positive effects of grape-derived products are expected primarily in the intestine. Some studies indeed confirm that grape-derived products have favorable effects on oxidative stress and inflammation, which is particularly beneficial for weaned piglets (see the section “Studies in weaned pigs”).

Although polyphenol concentrations in plasma and tissues are low, beneficial effects of grape-derived products in pigs and chickens have also been observed in plasma and tissues. As expected, these effects were less consistent across different studies. This variability may be due to the different forms (grape pomace, grape seeds, grape extracts, grape seed procyanidine extracts) and concentrations used in various studies. Thus, not only do polyphenol contents vary among preparations, but their bioavailability is also likely to be different. For example, it has been shown that hydrolyzable polyphenols have greater digestibility than condensed tannins in chicken [191].

From the comparison of studies on the effects of grape by-products in pigs and chickens on the one hand and cattle on the other, it becomes clear that the effects in cattle are less consistent and weaker overall. One possible cause for this could be differences in the bioavailability of polyphenols in the intestine. While polyphenols from grape-derived products directly reach the small intestine in monogastric animals, they undergo hydrolysis and biotransformation by the ruminal microbiota in ruminants [247]. The resulting aglycones and metabolites may then be partially absorbed in the small intestine [10]. Due to these transformations, the potential effects of polyphenols from grape-derived products are even more difficult to assess in ruminants compared to monogastric livestock.

As outlined in the sections above, several studies in pigs, broilers, and laying hens have shown that feeding grape-derived products improves feed efficiency. This effect is positive from both an economic perspective (feed costs) and an environmental perspective (resource efficiency, excretion of environmentally relevant substances). This beneficial effect is partly attributed to the inhibition of oxidative stress and inflammation. Many studies also indicate that polyphenols, particularly in monogastric livestock, have positive effects on the gut microbiota [248,249,250,251,252]. In recent years, the importance of the gut microbiota for animal health and performance has been increasingly recognized [152,253,254]. Therefore, the positive effects of grape-derived products on feed efficiency and animal performance may be at least partly due to their beneficial influence on the intestinal microbiota.

Studies conducted on growing-finishing pigs, broilers, and laying hens show that polyphenols can also protect lipids in products (meat, eggs) from peroxidation during storage. This effect is likely due to the incorporation of polyphenols into these products. Meat, in particular, is highly susceptible to oxidation due to the presence of myoglobin, which can induce lipid oxidation [255]. The polyphenols in these products can counteract the myoglobin-induced auto-oxidation of lipids through their radical-scavenging properties.

As outlined above, numerous studies suggest that grape-derived products possess antioxidant and anti-inflammatory properties. However, upon reviewing these studies, it becomes evident that many of them assessed only a limited number of antioxidant parameters (sometimes just the activity of one or a few antioxidant enzymes in plasma or serum) and anti-inflammatory markers (e.g., the expression of only a few cytokines in tissues). Oxidation products were mostly measured using TBARS in plasma or tissues. However, it should be noted that TBARS represent a relatively nonspecific parameter of lipid peroxidation, as thiobarbituric acid reacts with various aldehydes and breakdown products of proteins and carbohydrates [256]. The measurement of MDA, which is often assessed as an oxidation product in plasma, also relies on the TBARS assay. Therefore, further studies investigating the effects of grape-derived products with a more comprehensive assessment of antioxidant and anti-inflammatory parameters, as well as more specific lipid peroxidation markers, such as cholesterol oxidation products [257,258], would be desirable.

Studies in humans and rodent models have shown that oxidative stress and inflammation can also trigger ER stress, which is implicated in many diseases in humans, such as liver diseases, type 2 diabetes, and inflammatory diseases [259,260,261,262,263,264]. To date, ER stress has been less studied in farm animals. However, there is evidence that ER stress not only occurs in the liver and mammary glands of high-performing cows during the transition phase [114,228,265,266,267,268] but also in early-weaned piglets [269], lactating sows [270,271], and broilers suffering from heat stress [272]. It is known that polyphenols can mitigate ER stress in humans and rodent models [273,274,275,276]. However, the potential beneficial effects of grape-derived products on ER stress have been scarcely studied. It would be desirable to further explore the significance of ER stress in livestock in general and to investigate the potential benefits of grape-derived products in this context.

Overall, research suggests that grape-derived products primarily have beneficial effects on monogastric livestock (pigs, chickens) in terms of animal health (oxidative stress, inflammation) and, in many cases, performance (feed conversion ratio, weight gain). Moreover, utilizing grape-derived products, as by-products of winemaking, helps to reduce the environmental impact related to storage, transformation, and disposal. Based on the numerous studies reviewed, in which very different products were used in widely varying concentrations, it is difficult to provide clear recommendations for their practical usage in animal diets.

Despite their beneficial effects, grape-derived products also present certain limitations that restrict their use in animal feed. Polyphenols can bind to digestive enzymes in the intestine, leading to their inactivation. This may reduce the digestibility of nutrients, thereby negatively affecting feed efficiency [276]. However, studies on pigs have shown that concentrations of up to 9% grape pomace or 8% grape seeds in the diet do not adversely affect feed efficiency [158,160,166,277]. Similarly, broilers tolerated grape pomace at concentrations of up to 10% without impairing their feed conversion ratio [195,278,279,280]. Regarding grape seeds, inclusion levels up to 2% in the diet showed no negative effects on feed efficiency and even exhibited partial positive effects [179,281]. By contrast, broilers demonstrated lower tolerance to grape seed extract. A study found that administering 0.5% grape seed extract led to reduced protein digestibility and a deterioration in the feed conversion ratio [276]. Laying hens tolerated up to 6% grape pomace in their diet without performance losses or feed conversion ratio impairment [203,208,209]. However, supplementation with 1.5% grape seed reduced egg weight, even though the feed conversion ratio remained unaffected [207].

Overall, the findings indicate that monogastric farm animals can tolerate grape pomace at levels up to at least 6% without adverse performance effects. Similarly, most studies suggest good tolerance to grape seeds, which can also be included at levels of at least 2%. In contrast, grape seed extracts may negatively impact feed efficiency even at relatively low concentrations (below 1% in the diet).

Another aspect to consider when incorporating grape by-products into monogastric animals’ diets is that tannins—a subgroup of polyphenols present in grapes—can complex with cationic trace elements (e.g., iron, zinc, copper, and manganese) in the intestine, thereby reducing their bioavailability [282,283]. Feeding broilers a diet with 0.5% grape seed extract significantly reduced plasma concentrations of iron and zinc [276]. Similarly, a study on piglets found that a diet containing 1% grape seed and grape marc meal extract resulted in a significant reduction in hepatic zinc and copper levels [284]. Consequently, ensuring adequate trace element supplementation is essential when using grape by-products in pig and poultry diets.

In contrast, the data on cattle with respect to the antioxidant system and inflammation remain less clear. There are few indications that grape-derived products may exert positive effects on oxidative balance and inflammation in cattle. The use of grape by-products in cattle nutrition might be more interesting regarding their potential to modulate microbial fermentation in the rumen, which could lead to a reduction in methane emissions [211,212,213,285].

Overall, it can be concluded that grape by-products are valuable feedstuffs that can not only improve the health and performance of monogastric livestock but also contribute to environmental relief.

## Data Availability

No new data were created or analyzed in this study.

## Abbreviations

The following abbreviations are used in this manuscript:

APP	Acute-phase protein
APR	Acute-phase reaction
CAT	Catalase
COX2	Cyclooxygenase 2
ER	Endoplasmic reticulum
ERAD	ER-associated degradation
FGF21	Fibroblast growth factor 21
GPx	Glutathione peroxidase
GR	Glutathione reductase
GSH	Glutathione
GSPE	Grape seed proanthocyanidin extract
GST	Glutathione S-transferase
HO-1	Heme oxygenase-1
Ig	Immunoglobulin
IL	Interleukin
LPS	Lipopollysaccharide
MDA	Malondialdehyde
NF-kB	Nuclear factor kappa B
Nrf2	Nuclear factor-erythroid 2-related factor-2
PBMC	Peripheral blood mononuclear cells
ROS	Reactive oxygen species
SOD	Superoxide dismutase
TBARS	Thiobarbituric acid reactive substances
TGF	Transforming growth factor
TNF-α	Tumor necrosis factor α
UPR	Unfolded protein response
UPS	Ubiquitin–proteasome system

## References

[1] Martinez-Miro S., Tecles F., Ramon M., Escribano D., Hernandez F., Madrid J., Orengo J., Martinez-Subiela S., Manteca X., Ceron J.J. (2016). Causes, consequences and biomarkers of stress in swine: An update. BMC Vet. Res..

[2] Lauridsen C. (2019). From oxidative stress to inflammation: Redox balance and immune system. Poult. Sci..

[3] Kikusato M., Toyomizu M. (2023). Mechanisms underlying the effects of heat stress on intestinal integrity, inflammation, and microbiota in chickens. J. Poult. Sci..

[4] Tufarelli V., Colonna M.A., Losacco C., Puvaca N. (2023). Biological health markers associated with oxidative stress in dairy cows during lactation period. Metabolites.

[5] Lavecchia T., Rea G., Antonacci A., Giardi M.T. (2013). Healthy and adverse effects of plant-derived functional metabolites: The need of revealing their content and bioactivity in a complex food matrix. Crit. Rev. Food Sci. Nutr..

[6] Mei H., Li Y., Wu S., He J. (2024). Natural plant polyphenols contribute to the ecological and healthy swine production. J. Anim. Sci. Biotechnol..

[7] Chen J., Huang Z., Cao X., Zou T., You J., Guan W. (2022). Plant-derived polyphenols in sow nutrition: An update. Anim. Nutr..

[8] Al Rharad A., El Aayadi S., Avril C., Souradjou A., Sow F., Camara Y., Hornick J.L., Boukrouh S. (2025). Meta-Analysis of Dietary Tannins in Small Ruminant Diets: Effects on Growth Performance, Serum Metabolites, Antioxidant Status, Ruminal Fermentation, Meat Quality, and Fatty Acid Profile. Animals.

[9] Boukrouh S., Noutfia A., Moula N., Avril C., Hornick J.-L., Chentouf M., Cabaraux J.-F. (2023). Effects of *Sulla flexuosa* Hay as Alternative Feed Resource on Goat’s Milk Production and Quality. Animals.

[10] Gessner D.K., Ringseis R., Eder K. (2017). Potential of plant polyphenols to combat oxidative stress and inflammatory processes in farm animals. J. Anim. Physiol. Anim. Nutr..

[11] Saracila M., Panaite T.D., Papuc C.P., Criste R.D. (2021). Heat stress in broiler chickens and the effect of dietary polyphenols, with special reference to willow (*Salix* spp.) bark supplements—A review. Antioxidants.

[12] Serra V., Salvatori G., Pastorelli G. (2021). Dietary polyphenol supplementation in food producing animals: Effects on the quality of derived products. Animals.

[13] Yang C., Han Y., Tian X., Sajid M., Mehmood S., Wang H., Li H. (2024). Phenolic composition of grape pomace and its metabolism. Crit. Rev. Food. Sci. Nutr..

[14] Prata C., Zalambani C., Rossi F., Rossello S., Cerchiara T., Cappadone C., Malucelli E. (2025). Nutrients and nutraceuticals from *Vitis vinifera* L. pomace: Biological activities, valorization, and potential application. Nutrients.

[15] Karastergiou A., Gancel A.-L., Jourdes M., Teissedre P.-L. (2024). Valorization of grape pomace: A review of phenolic composition, bioactivity, and therapeutic potential. Antioxidants.

[16] Costa M.M., Alfaia C.M., Lopes P.A., Pestana J.M., Prates J.A. (2022). Grape by-products as feedstuff for pig and poultry. Animals.

[17] Brenes A., Viveros A., Chamorro S., Arija I. (2016). Use of polyphenol-rich grape by-products in monogastric nutrition. A review. Anim. Feed Sci. Technol..

[18] Sies H. (1997). Oxidative stress: Oxidants and antioxidants. Exp. Physiol..

[19] Lykkesfeldt J., Svendsen O. (2007). Oxidant and antioxidants in disease: Oxidative stress in farm animals. Vet. J..

[20] Sordillo L.M., Aitken S.L. (2009). Impact of oxidative stress on the health and immune function of dairy cattle. Vet. Immunol. Immunopathol..

[21] Abuelo A., Hernandez J., Benedito L., Castillo C. (2015). The importance of the oxidative status of dairy cattle in the periparturient period: Revisiting antioxidant supplementation. J. Anim. Physiol. Anim. Nutr..

[22] Celi P., Gabai G. (2015). Oxidant/antioxidant balance in the animal nutrition: The role of protein oxidation. Front. Vet. Sci..

[23] Evans P., Halliwell B. (2001). Micronutrients: Oxidant/antioxidant status. Br. J. Nutr..

[24] Ito H., Kurokawa H., Matsui H. (2021). Mitochondrial reactive oxygen species and heme, non-heme iron metabolism. Arch. Biochem. Biophys..

[25] Ayer A., Fazakerley D.J., James D.E., Stocker R. (2022). The role of mitochondrial reactive oxygen species in insulin resistance. Free Radic. Biol. Med..

[26] Belambri S.A., Rolas L., Raad H., Hurtado-Nedelec M., Dang P.M.-C., El-Benna J. (2018). NADPH oxidase activation in neutrophils: Role of the phosphorylation of its subunits. Eur. J. Clin. Investig..

[27] Manoharan R.R., Prasad A., Pospisil P., Kzhyshkowska J. (2024). ROS signalling in innate immunity via oxidative protein modifications. Front. Immunol..

[28] Halliwell B. (1996). Oxidative stress, nutrition and health. Experimental strategies for optimization of nutritional antioxidant intake in humans. Free Rad. Res..

[29] Halliwell B., Gutteridge J.M.C. (1990). Role of free radicals and catalytic metal ions in human disease. Methods Enzymol..

[30] Gutteridge J.M.C. (1994). Biological origin of free radicals, and mechanisms of antioxidant protection. Chem.-Biol. Interact..

[31] Raederstorff D., Wyss A., Calder P.C., Weber P., Eggersdorfer M. (2015). Vitamin E function and requirements in relation to PUFA. Br. J. Nutr..

[32] Javouhey-Donzel A., Guenot L., Maupoil V., Rochette L., Rocquelin G. (1993). Rat vitamin E status and heart lipid peroxidation: Effect of dietary alpha-linolenic acid and marine n-3 fatty acids. Lipids.

[33] Eder K., Grünthal G., Kluge H., Hirche F., Spilke J., Brandsch C. (2005). Concentrations of cholesterol oxidation products in raw, heat-processed and frozen-stored meat of broiler chickens fed diets differing in the type of fat and vitamin E concentrations. Br. J. Nutr..

[34] Eder K., Kirchgessner M. (1998). The effect of dietary vitamin E supply and a moderately oxidized oil activities of hepatic lipogenic enzymes in rats. Lipids.

[35] Liu J.F., Huang C.J. (1996). Dietary oxidised frying oil enhances tissue α-toccopherol depletion and radioisotope tracer excretion in vitamin E-deficient rats. J. Nutr..

[36] Keller U., Brandsch C., Eder K. (2004). Supplementation of vitamins C and E increases the vitamin E status but does not prevent the formation of oxysterols in the liver of guinea pigs fed an oxidised fat. Eur. J. Nutr..

[37] Banerjee B.D., Seth V., Ahmed R.S. (2001). Pesticide-induced oxidative stress: Perspectives and trends. Rev. Environ. Health.

[38] Alpsoy L., Yalvac M.E. (2011). Key roles of vitamins A, C, and E in aflatoxin B1-induced oxidative stress. Vitam. Horm..

[39] Yanagi T. (2007). Molecular mechanism of phase I and phase II drug-metabolizing enzymes: Implications for etoxification. Int. Rev. Cytol..

[40] Omura T. (1999). Forty years of cytochrome P450. Biochem. Biophys. Res. Commun..

[41] Goeptar A.R., Scheerens H., Vermeulen N.P.E. (1995). Oxygen and xenobiotic reductase activities of cytochrome P450. Crit. Rev. Toxicol..

[42] Lennicke C., Cocheme H.M. (2021). Redox metabolism: ROS as specific molecular regulators of cell signalling and function. Cell.

[43] Halliwell B. (2007). Biochemistry of oxidative stress. Biochem. Soc. Trans..

[44] Halliwell B. (2024). Understanding mechanisms of antioxidant action in health and disease. Nat. Rev. Mol. Cell Biol..

[45] Kim J., Cha Y.-N., Surh Y.-J. (2010). A protective role of nuclear factor-erythroid-2-related factor-2 (Nrf2) in inflammatory disorders. Mutat. Res..

[46] Lu M.C., Ji J.A., Jiang Z.Y., You Q.D. (2016). The Keap-1-Nrf2-ARE pathway as a potential preventive and therapeutic target: An update. Med. Res. Rev..

[47] Salman S., Paulet V., Hardonniere K., Kerdine-Römer S. (2025). The role of NRF2 transcription factor in inflammatory skin diseases. BioFactors.

[48] Jomova K., Raptova R., Alomar S.Y., Alwasel S.H., Nepovimova E., Kuca K., Valko M. (2023). Reactive ocygen species, toxicity, oxidative stress, and antioxidants: Chronic diseases and aging. Arch. Toxicol..

[49] Sidker M.M., Li X., Akumwami S., Labony S.A. (2025). Reactive oxygene species: Role in pathophysiology, and mechanism of endogenous and dietary antioxidants during oxidative stress. Chonnam. Med. J..

[50] Boukrouh S., Noutfia A., Moula N., Avril C., Louvieaux J., Hornick J.-L., Chentouf M., Cabaraux J.-F. (2023). Ecological, Morpho-Agronomical, and Nutritional Characteristics of *Sulla flexuosa* (L.) Medik. Ecotypes. Sci. Rep..

[51] Tapia P.C. (2006). Subletal mitochondrial stress with an attendant stoichiometric augmentation of reactive oxygen species may precipitate many of the beneficial alterations in cellular physiology produced by caloric restriction, intermittent fasting, exercise and dietary phytonutrients: “Mitohormesis” for health and vitality. Med. Hypotheses.

[52] Kasai S., Shimizu S., Tatara Y., Mimura J., Itoh K. (2020). Regulation of Nrf2 by mitochondrial reactive oxygen species in physiology and pathology. Biomolecules.

[53] Min S.H., Kang G.M., Park J.W., Kim M.S. (2024). Beneficial effects of low-grade mitochondrial stress on metabolic diseases and aging. Yonsei Med. J..

[54] Ristow M., Schmeisser S. (2011). Extending life span by increasing oxidative stress. Free Radic. Biol. Med..

[55] Ristow M., Zarse K. (2010). How increased oxidative stress promotes longevity and metabolic health: The concept of mitochondrial hormesis (mitohormesis). Exp. Gerontol..

[56] Capozzi A., Saucier C., Bisbal C., Lambert K. (2022). Grape polyphenols in the treatment of human skeletal muscle damage due to inflammation and oxidative stress during obesity and aging: Early outcomes and promises. Molecules.

[57] Bertoni G., Trevisi E., Han X., Bionaz M. (2008). Effects of inflammatory conditions on liver activity in puerperium period and consequences for performance in dairy cows. J. Dairy Sci..

[58] Newton K., Dixit V.M. (2012). Signaling in innate immunity and inflammation. Cold Spring Harb. Perspect. Biol..

[59] Bradford B.J., Yuan K., Farney J.K., Mamedova L.K., Carpenter A.J. (2015). Invited review: Inflammation during the transition to lactation: New adventures with an old flame. J. Dairy Sci..

[60] Vendrame S., Klimis-Zacas D. (2015). Anti-inflammatory effect of anthocyanins via modulation of nuclear factor-κB and mitogen-activated protein kinase signaling cascades. Nutr. Rev..

[61] Michalak K.P., Michalak A.Z. (2025). Understanding chronic inflammation: Couplings between cytokines, ROS, NO, Ca_i_^2+^, HIF-1α, Nrf2 and autophagy. Front. Immunol..

[62] Schmidt-Arras D., Rose-John S. (2016). IL-6 pathway in the liver: From physiopathology to therapy. J. Hepatol..

[63] Schrödl W., Büchler R., Wendler S., Reinhold P., Muckova P., Reindl J., Rhode H. (2016). Acute phase proteins as promising biomarkers: Perspectives and limitations for human and veterinary medicine. Proteom. Clin. Appl..

[64] Venteclef N., Jakobsson T., Steffensen K.R., Treuter E. (2011). Metabolic nuclear receptor signaling and the inflammatory acute phase response. Trends Endocrinol. Metabol..

[65] Ceciliani F., Ceron J.J., Eckersall P.D., Sauerwein H. (2012). Acute phase proteins in ruminants. J. Proteom..

[66] Saco Y., Bassols A. (2023). Acute phase proteins in cattle and swine: A review. Vet. Clin. Pathol..

[67] Jacobsen N., Weber N.R., Larsen I., Pedersen K.S. (2024). Diagnostic utility of acute phase proteins and their ability to guide antibiotic usage in pigs, horses, and cattle: A mapping review. Acta Vet. Scand..

[68] Petersen H.H., Nielsen J.P., Heegard P.M.H. (2004). Application of acute phase protein measurements in veterinary clinical chemistry. Vet. Res..

[69] Polepalle T., Moogala S., Boggarapu S., Pesala D.S., Palagi F.B. (2015). Acute phase proteins and their role in periodontitis: A review. J. Clin. Diagn. Res..

[70] Bertoni G., Trevisi E. (2013). Use of liver activity index and other metabolic variables in the assessment of metabolic health in dairy herds. Vet. Clin. N. Am. Food Anim. Pract..

[71] Burfeind K.G., Michaelis K.A., Marks D.L. (2015). The central role of hypothalamic inflammation in the acute illness response and cachexia. Semin. Cell Dev. Biol..

[72] Dwarkasing J.T., Marks D.L., Witkamp R.F., van Norren K. (2016). Hypothalamic inflammation and food intake regulation during chronic illness. Peptides.

[73] Karrow N.A. (2006). Activation of the hypothalamic-pituitary-adrenal axis and autonomic nervous system during inflammation and altered programming of the neuroendocrine-immune axis during fetal and neonatal development: Lessons learned from the model inflammagen, lipopolysaccharide. Brain Behav. Immun..

[74] Bowen T.S., Schuler G., Adams V. (2015). Skeletal muscle wasting in cachexia and sarcopenia: Molecular pathophysiology and impact of exercise training. J. Cachexia Sarcop. Muscle.

[75] Klasing K.C., Johnstone B.J. (1991). Monokines in growth and development. Poult. Sci..

[76] Johnson R.W. (1997). Inhibition of growth by pro-inflammatory cytokines: An integrated view. J. Anim. Sci..

[77] Andus T., Bauer J., Gerok W. (1991). Effects of cytokines on the liver. Hepatology.

[78] Thaler J.P., Choi S.J., Schwartz M.W., Wisse B.E. (2010). Hypothalamic inflammation and energy homeostasis: Resolving the paradox. Front. Neuroendocrinol..

[79] Langhans W. (2000). Anorexia of infection: Current prospects. Nutrition.

[80] Plata-Salaman C.R. (1996). Anorexia during acute and chronic disease. Nutrition.

[81] Exton M.S. (1997). Infection-induced anorexia: Active host defence strategy. Appetite.

[82] Beisel W.R. (1984). Metabolic effects of infection. Prog. Food Nutr..

[83] Ganz T. (2018). Iron and infection. Int. J. Hematol..

[84] Parrow N.L., Fleming R.E., Minnick M.F. (2013). Sequestration and scavenging of iron in infection. Infect. Immunol..

[85] Andrews S.C., Robinson A.K., Rodriguez-Quinones F. (2003). Bacterial iron homeostasis. FEMS Microbiol. Rev..

[86] Drakesmith H., Prentice A.M. (2012). Hepcidin and the iron-infection axis. Science.

[87] Murray M.J., Murray B.H. (1979). Anorexia of infection as a host defense. Am. J. Clin. Nutr..

[88] Cnop M., Foufelle F., Velloso L.A. (2012). Endoplasmic reticulum stress, obesity and diabetes. Trends Mol. Med..

[89] Marciniak S.J., Ron D. (2006). Endoplasmic reticulum stress signaling in disease. Physiol. Rev..

[90] Ron D., Walter P. (2007). Signal integration in the endoplasmic reticulum unfolded protein response. Nat. Rev. Mol. Cell Biol..

[91] Rutkowski D.T., Kaufman R.J. (2004). A trip to the ER: Coping with stress. Trends Cell Biol..

[92] Breckenridge D.G., Germain M., Mathai J.P., Nguyen M., Shore G.C. (2003). Regulation of apoptosis by endoplasmic reticulum pathways. Oncogene.

[93] Obaseki I., Ndolo C.C., Adedeji A.A., Popoola H.O., Kravats A.N. (2025). The structural and functional dynamics of BiP and Grp94: Opportunities for therapeutic discovery. Trends Pharmacol. Sci..

[94] Salminen A., Kaarniranta K., Kauppinen A. (2017). Integrated stress response stimulates FGF21 expression: Systemic enhancer of longevity. Cell. Signal..

[95] Shimizu M., Sato R. (2022). Endocrine fibroblast growth factors in relation to stress signalling. Cells.

[96] Carriquiry M., Weber W.J., Fahrenkrug S.C., Crooker B.A. (2009). Hepatic gene expression in multiparous Holstein cows treated with bovine somatotropin and fed n-3 fatty acids in early lactation. J. Dairy Sci..

[97] Schoenberg K.M., Giesy S.L., Harvatine K.J., Waldron M.R., Cheng C., Kharitonenkov A., Boisclair Y.R. (2011). Plasma FGF21 is elevated by the intense lipid mobilization of lactation. Endocrinology.

[98] Schlegel G., Ringseis R., Keller J., Schwarz F.J., Windisch W., Eder K. (2013). Expression of fibroblast growth factor 21 in the liver of dairy cows in the transition period and during lactation. J. Anim. Physiol. Anim. Nutr..

[99] Drong C., Bühler S., Frahm J., Hüther L., Meyer U., von Soosten D., Gessner D.K., Eder K., Sauerwein H., Dänicke S. (2017). Effects of body condition, monensin, and essential oils on ruminal lipopolysaccharide concentration, inflammatory markers, and endoplasmatic reticulum stress of transition dairy cows. J. Dairy Sci..

[100] Wang J., Zhu X., She G., Kong Y., Guo Y., Wang Z., Liu G., Zhao B. (2018). Serum hepatokines in dairy cows: Periparturient variation and changes in energy-related metabolic disorders. BMC Vet. Res..

[101] Rosenbaum S., Ringseis R., Most E., Hillen S., Becker S., Erhardt G., Reiner G., Eder K. (2013). Genes involved in carnitine synthesis and carnitine uptake are up-regulated in the liver of sows during lactation. Acta Vet. Scand..

[102] Eder K., Gessner D.K., Ringseis R. (2021). Fibroblast growth factor 21 in dairy cows: Current knowledge and potential relevance. J. Anim. Sci. Biotechnol..

[103] Kim O.K., Jun W., Lee J. (2015). Mechanism of ER stress and inflammation for hepatic insulin resistance in obesity. Ann. Nutr. Metab..

[104] Ajoolabady A., Lebeaupin C., Wu N.N., Kaufman R.J., Ren J. (2023). ER stress and inflammation cross talk in obesity. J. Med. Res. Rev..

[105] Drong C., Meyer U., von Soosten D., Frahm J., Rehage J., Breves G., Dänicke S. (2016). Effect of monensin and essential oils on performance and energy metabolism of transition dairy cows. J. Anim. Physiol. Anim. Nutr..

[106] Schuh K., Sadri H., Häussler S., Webb L.A., Urh C., Wagner M., Koch C., Frahm J., Dänicke S., Dusel G. (2019). Comparison of performance and metabolism from late pregnancy to early lactation in dairy cows with elevated *v*. normal body condition at dry-off. Animal.

[107] Casaro S., Perez-Baez J., Bisinotto R.S., Chebel R.C., Prim J.G., Gonzalez T.D., Carvalho Gomes G., Tao S., Toledo I.M., do Amaral B.C. (2024). Association between prepartum body condition score and prepartum and postpartum dry matter intake and energy balance in multiparous Holstein cows. J. Dairy Sci..

[108] Bernabucci U., Ronchi B., Lacetera N., Nardone A. (2005). Influence of body condition score on relationships between metabolic status and oxidative stress in periparturient dairy cows. J. Dairy Sci..

[109] Alharti A., Zhou Z., Lopreiato V., Trevisi E., Loor J.J. (2018). Body condition score prior to parturition is associated with plasma and adipose tissue biomarkers of lipid metabolism and inflammation in Holstein cows. J. Anim. Sci. Biotechnol..

[110] Celik C., Lee S.Y.T., Yap W.S., Tibault G. (2023). Endoplasmic reticulum stress and lipids in health and disease. Prog. Lipid Res..

[111] Oakes S.A., Papa F.R. (2015). The role of endoplasmic reticulum stress in human pathology. Annu. Rev. Pathol..

[112] Alotaibi G., Alkhammash A. (2025). Pharmacological landscape of endoplasmc reticulum stress: Uncovering therapeutic avenues for metabolic diseases. Eur. J. Pharmacol..

[113] Ringseis R., Gessner D.K., Eder K. (2015). Molecular insights into the mechanisms of liver-associated diseases in early-lactating dairy cows: Hypothetical role of endoplasmic reticulum stress. J. Anim. Physiol. Anim. Nutr..

[114] Gessner D.K., Schlegel G., Ringseis R., Schwarz F.J., Eder K. (2014). Up-regulation of endoplasmic reticulum stress induced genes of the unfolded protein response in the liver of periparturient dairy cows. BMC Vet. Res..

[115] Rodriguez-Perez C., Garcia-Villanova B., Guerra-Hernandez E., Verardo V. (2019). Grape seed proanthocyanidins: An overview of in vivo bioactivity in animal models. Nutrients.

[116] Pop R.M., Boarescu P.M., Bocsan C.I., Gherman M.L., Chedea V.S., Jianu E.M., Rosian S.H., Boarescu I., Ranga F., Tomoiaga L.L. (2025). Anti-Inflammatory and Antioxidant Effects of White Grape Pomace Polyphenols on Isoproterenol-Induced Myocardial Infarction. Int. J. Mol. Sci..

[117] Bocsan I.C., Magureanu D.C., Pop R.M., Levai A.M., Macovei S.O., Patrasca I.M., Chedea V.S., Buzoianu A.D. (2022). Antioxidant and anti-inflammatory actions of polyphenols from red and white grape pomace in ischemic heart disease. Biomedicines.

[118] Taladrid D., Rebollo-Hernanz M., Martin-Cabrejas M.A., Moreno-Arribas M.V., Bartolome B. (2023). Grape pomace as a cardiometabolic health-promoting ingredient: Activity in the intestinal environment. Antioxidants.

[119] Calabrese V., Cornelius C., Dinkova-Kostova A.T., Iavicoli I., Di Paola R., Koverech A., Cuzzocrea S., Rizzarelli E., Calabrese E.J. (2012). Cellular stress responses, hormetic phytochemicals and vitagenes in aging and longevity. Biochim. Biophys. Acta.

[120] Murakami A. (2014). Dose-dependent functionality and toxicity of green tea polyphenols in exterimental rodents. Arch. Biochem. Biophys..

[121] Jomova K., Alomar S.Y., Valko R., Liska J., Nepovimova E., Kuca K., Valko M. (2025). Flavonoids and their role in oxidative stress, inflammation and human diseases. Chem. Biol. Interact..

[122] Liu M., Liu S., Lin Z., Chen X., Jiao Q., Du X., Jiang H. (2025). Targeting the Interplay between Autophagy and the Nrf2 Pathway in Parkinson’s Disease with Potential Therapeutic Implications. Biomolecules.

[123] Lee J., Giordano S., Zhang J. (2012). Autophagy, mitochondria and oxidative stress: Cross-talk and redox signalling. Biochem. J..

[124] Kim H.S., Montana V., Jang H.J., Parpura V., Kim J.A. (2013). Epigallocatechin gallate (EGCG) stimulates autophagy in vascular endothelial cells: A potential role for reducing lipid accumulation. J. Biol. Chem..

[125] Pandey P., Lakhanpal S., Mahmood D., Kang H.N., Kim B., Kang S., Choi J., Choi M., Pandey S., Bhat M. (2025). An updated review summarizing the anticancer potential of flavonoids via targeting NF-KB pathway. Front. Pharmacol..

[126] Terra X., Pallares V., Ardevol A., Blade C., Fernandez-Larrea J., Pujadas G., Salvado J., Arola L., Blay M. (2011). Modulatory effect of grape-seed procyanidins on local and systemic inflammation in diet-induced obesity rats. J. Nutr. Biochem..

[127] Liu X., Liu M., Liu X., Lan Z. (2018). Grape seed proanthocyanidin extract supplementation affects exhaustive exercise-induced fatigue in mice. Food Nutr. Res..

[128] Crescenzi E., Leonardi A., Pacifico F. (2024). NF-KB in thyroid cancer: An update. Int. J. Mol. Sci..

[129] Jiang Y., Zhang J., Shi C., Li X., Jiang Y., Mao R. (2023). NF-KB: A mediator that promotes or inhibits angiogenesis in human diseases?. Expert Rev. Mol. Med..

[130] Keleku-Lukwete N., Suzuki M., Yamamoto M. (2018). An overview of the advantages of KEAP1-NRF2 system activation during inflammatory disease treatment. Antioxid. Redox Signal..

[131] Saha S., Buttari B., Panieri E., Profumo E., Saso L. (2020). An overviewof Nrf2 signaling pathway and its role in inflammation. Molecules.

[132] Zhang J.J., Ni P., Song Y., Gao M.J., Guo X.Y., Zhao B.Q. (2024). Effective protective mechanisms of HO-1 in diabetic complications: A narrative review. Cell Death Discov..

[133] Chuang C.C., McIntosh M.K. (2011). Potential mechanisms by which polyphenol-rich grapes prevent obesity mediated inflammation and metabolic diseases. Annu. Rev. Nutr..

[134] Food and Agriculture Organization (FAO) “FAOSTAT”. https://www.fao.org/faostat/en/#home.

[135] Tangney C.C., Rasmussen H.E. (2013). Polyphenols, inflammation, and cardiovascular disease. Curr. Atheroscl. Rep..

[136] Zhou F., Deng S., Luo Y., Liu Z., Liu C. (2025). Research progress on the protective effect of green tea polyphenol (-)-epigallocatechin-3-gallate (EGCG) on the liver. Nutrients.

[137] Uifalean A., Schneider S., Ionescu C., Lalk M., Iuga C.A. (2015). Soy isoflavones and breast cancer cell lines: Molecular mechanisms and future perspectives. Molecules.

[138] Chedea V.S., Macovei S.O., Bocsan I.C., Măgureanu D.C., Levai A.M., Buzoianu A.D., Pop R.M. (2022). Grape Pomace Polyphenols as a Source of Compounds for Management of Oxidative Stress and Inflammation-A Possible Alternative for Non-Steroidal Anti-Inflammatory Drugs?. Molecules.

[139] Castellanos-Gallo L., Ballinas-Casarrubias L., Espinoza-Hicks J.C., Hernández-Ochoa L.R., Muñoz-Castellanos L.N., Zermeño-Ortega M.R., Borrego-Loya A., Salas E. (2022). Grape pomace valorization by extraction of phenolic polymeric pigments: A review. Processes.

[140] Winkler A., Weber F., Ringseis R., Eder K., Dusel G. (2015). Determination of polyphenol and crude nutrient content and nutrient digestibility of dried and ensiled white and red grape pomace cultivars. Arch. Anim. Nutr..

[141] Makris D.P., Boskou G., Andrikopoulos N.K. (2007). Recovery of antioxidant phenolics from white vinification solid by-products employing water/ethanol mixtures. Bioresour. Technol..

[142] Ky I., Teissedre P.L. (2015). Characterisation of Mediterranean grape pomace seed and skin extracts: Polyphenolic content and antioxidant activity. Molecules.

[143] Gonzalez-Centeno M.R., Jourdes M., Femenia A., Siml S., Rossello C., Teissedre P.-L. (2013). Characterization of polyphenols and antioxidant properties of white grape pomace byproducts (*Vitis vinifera* L.). J. Agric. Food Chem..

[144] De la Cerda-Carrasco A., Lopez-Solis R., Nunez-Kalasic H., Pena-Neira A., Obreque-Slier E. (2015). Phenolic composition and antioxidant capacity of pomaces from four grape varieties (*Vitis vinifera* L.). J. Sci. Food Agric..

[145] Ky I., Lorrain B., Kolbas N., Crozier A., Teissedre P.L. (2014). Wine by-products: Phenolic characterization and antioxidant activity evaluation of grapes and grape pomaces from six different French grape varieties. Molecules.

[146] De Bellis P., Maggiolino A., Albana C., De Palo P., Blando F. (2022). Ensiling grape pomace with and without addition of *Lactiplantibacillus plantarum* strain: Effect on polyphenols and microbiological characteristics, in vitro nutrient apparent digestibility, and gas emission. Front. Vet. Sci..

[147] Kammerer D., Claus A., Carle R., Schieber A. (2024). Polyphenol screening of pomace from red and white grape varities (*Vitis vinifera* L.) by HPLC-DAD-MS/MS. J. Agric. Food Chem..

[148] Tapia-Quiros P., Montenegro-Landivar M.F., Reig M., Vecino X., Cortina J.L., Saurina J., Granados M. (2022). Recovery of agri-food by products: The olive oil and winery industries cases. Foods.

[149] Guaita M., Bosso A. (2019). Polyphenolic characterization of grape skins and seeds of four Italian red cultivars at harvest and fermentative maceration. Foods.

[150] Tang G.Y., Zhao C.N., Liu Q., Feng X.L., Xu X.Y., Cao S.Y., Meng X., Li S., Gan R.Y., Li H.B. (2018). Potential of grape wastes as a natural source of bioactive compounds. Molecules.

[151] Pie S., Lalles J.P., Blazy F., Laffitte J., Seve B., Oswald I.P. (2004). Weaning is associated with an upregulation of expression of inflammatory cytokines in the intestine of piglets. J. Nutr..

[152] Kiernan D.P., O’Doherty J.V., Sweeney T. (2023). The effect of prebiotic supplements on the gastrointestinal microbiota and associated health parameters in pigs. Animals.

[153] Gessner D.K., Fiesel A., Most E., Dinges J., Wen G., Ringseis R., Eder K. (2013). Supplementation of a grape seed and grape marc meal extract decreases activities of the oxidative stress-responsive transcription factors NF-κB and Nrf2 in the duodenal mucosa of pigs. Acta Vet. Scand..

[154] Fiesel A., Gessner D.K., Most E., Eder K. (2014). Effects of dietary polyphenol-rich plant products from grape or hop on pro-inflammatory gene expression in the intestine, nutrient digestibility and faecal microbiota of weaned pigs. BMC Vet. Res..

[155] Wei X., Li L., Yan H., Li Q., Gao J., Hao R. (2022). Grape seed procyanidins improve intestinal health by modulating gut microbiota and enhancing intestinal antioxidant capacity in weaned piglets. Livest. Sci..

[156] Hao R.R., Li Q.H., Zhao J.Q., Li H.F., Wang W.W., Gao J.J. (2015). Effects of rape seed procyanidins on growth performance, immune function and antioxidative capacity in weaned piglets. Livest. Sci..

[157] Han M., Song P., Huang C., Rezaei A., Farrar S., Brown M., Ma X. (2016). Dietary grape seed procyanidins (GSPs) improve weaned intestinal microbiota and mucosal barrier using a piglet model. Oncotarget.

[158] Chedea V.S., Palade L.M., Marin D.E., Pelmus R.S., Habeanu M., Rotar M.C., Gras M.A., Pistol G.C., Taranu I. (2018). Intestinal Absorption and Antioxidant Activity of Grape Pomace Polyphenols. Nutrients.

[159] Rajkovic E., Schwarz C., Kapsamer S.B., Schedle K., Reisinger N., Emsenhuber C., Ocelova V., Roth N., Frieten D., Dusel G. (2022). Evaluation of a dietary grape extract on oxidative status, intestinal morphology, plasma acute-phase proteins and inflammation parameters of weaning piglets at various points of time. Antioxidants.

[160] Kafantaris I., Stagos D., Kotsampasi B., Hatzis A., Kypriotakis A., Gerasopoulos K., Makri S., Goutzourelas N., Mitsagga C., Giavasis I. (2018). Grape pomace improves performance, antioxidant status, fecal microbiota and meat quality of piglets. Animal.

[161] Pistol G.C., Marin D.E., Bulgaru V.C., Anghel A.C., Saracila M., Vlassa M., Filip M., Taranu I. (2023). Grape seed meal by-product is able to counteract oxidative stress induced by lipopolysaccharide and dextran sulphate in IPEC cells and piglets after weaning. PLoS ONE.

[162] Gessner D.K., Bonarius M., Most E., Fiesel A., Eder K. (2017). Effects of polyphenol-rich plant products from grape or hop as feed supplements on the expression of inflammatory, antioxidative, cytoprotective and endoplasmic reticulum stress-related genes and the antioxidative status in the liver of piglets. J. Anim. Physiol. Anim. Nutr..

[163] Taranu I., Habeanu M., Gras M.A., Pistol G.C., Lefter N., Palade M., Ropota M., Chedea V.S., Marin D.E. (2018). Assessment of the effect of grape seed cake inclusion in the diet of healthy fattening-finishing pigs. J. Physiol. Anim. Nutr..

[164] Pistol G.C., Bulgaro V.C., Grosu I.A., Ciupitu A.-M., Taranu I. (2024). The effect of a diet containing grape seed meal on inflammatory and antioxidant markers in spleen of weaned piglets. Arch. Zootechn..

[165] Park J.C., Lee S.H., Hong J.K., Cho J.H., Kim I.H., Park S.K. (2014). Effect of dietary supplementation of procyanidin on growth performance and immune response in pigs. Asian Aust. J. Anim. Sci..

[166] Wang R., Yu H., Fang H., Jin Y., Zhao Y., Shen J., Zhou C., Li R., Wang J., Fu Y. (2020). Effects of dietary grape pomace on the intestinal microbiota and growth performance of weaned piglets. Arch. Anim. Nutr..

[167] Wu Y., Ma N., Song P., He T., Levesque C., Bai Y., Zhang A., Ma X. (2019). Grape seed proanthocyanidin affects lipid metabolism via changing gut microflora and enhancing propionate production in weaned pigs. J. Nutr..

[168] Zheng Y., Li Y., Yu B., Huang Z., Luo Y., Zheng P., Mao X., Yu J., Tan H., Luo J. (2024). Grape seed proanthocyanidins improves growth performance, antioxidative capacity, and intestinal microbiota in growing pigs. Front. Microbiol..

[169] Taranu I., Habeanu M., Gras M.A., Pistol G.C., Lefter N., Palade M., Ropota M., Chedea V.S., Marin D.E. (2017). Effect of xenobiotic compounds from grape waste on liver function and oxidative status in pigs. Arch. Zootech..

[170] Horodincu L., Proca A.C., Slencu B.G., Trifan A., Pavel G., Solcan G., Solcan C. (2025). Modulating effects of grape pomace on the intestinal antioxidative and inflammatory status in fattening pigs. Agriculture.

[171] Yan L., Kim I.H. (2011). Effect of dietary grape pomace fermented by Saccharomyces boulardii on the growth performance, nutrient digestibility and meat quality in finishing pigs. Asian Austr. J. Anim. Sci..

[172] Trombetta F., Fruet A.P.B., Stefanello F.S., Fonseca P.A.F., De Souza A.N.M., Tonetto C.J., Rosado Junior A.G., Nörnberg J.L. (2019). Effects of the dietary inclusion of linseed oil and grape pomace on weight gain, carcass characteristics, and met quality of swine. Int. Food Res. J..

[173] Tian X., Li D., Zhao X., Xiao Z., Sun J., Yuan T., Wang Y., Zuo X., Yang G., Yu T. (2023). Dietary grape pomace extract supplementation improved meat quality, antioxidant capacity, and immune performance in finishing pigs. Front. Microbiol..

[174] Wu H., Bak K.H., Goran G.V., Tatiyaborntham N. (2024). Inhibitory mechanisms of polyphenols on heme protein-mediated lipid oxidation in muscle food: New insights and advances. Crit. Rev. Food Sci. Nutr..

[175] Zhu W., Han M., Bu Y., Li X., Yi S., Xu Y., Li J. (2024). Plant polyphenols regulating myoglobin oxidation and color stability in red meat and certain fish: A review. Crit. Rev. Food Sci. Nutr..

[176] Wang X., Jiang G., Kebreab E., Yu Q., Li J., Zhang X., He H., Fang R., Dai Q. (2019). Effect of dietary grapeseed polyphenols supplementation during late gestation and lactation on antioxidant status in serum and immunoglobulin content in colostrum of multiparous sows. J. Anim. Sci..

[177] Wang M., He Z., Xiong Z., Liu H., Zhou X., He J. (2024). Effects of dietary supplementation of grape seed extract in comparison with excessive level of vitamin E on growth performance and antioxidant function of broilers. Anim. Biotechnol..

[178] Noor S., Al-Mashhdani E., Kadhim H. (2022). Effects of grape seed powder on productive performance, lipid profile and total bacteria in duodenum and ceca of broiler chickens. Arch. Razi Inst..

[179] Gungor E., Altop A., Erener G. (2021). Effect of raw and fermented grape seed on growth performance, antioxidant capacity, and cecal microflora in broiler chickens. Animal.

[180] Cao G., Zeng X., Liu J., Xiang Z., Wang Y., Tao F., Yang C. (2020). Change of serum metabolome and cecal microflora in broiler chickens supplemented with grape seed extract. Front. Immunol..

[181] Rajput S.A., Sun L., Zhang N.Y., Khalil M.M., Ling Z., Chong L., Wang S., Rajput I.R., Bloch D.M., Khan F.A. (2019). Grape seed proanthocyanidin extract alleviates aflatoxin B1-induced immunotoxicity and oxidative stress via modulation of NF-κB and Nrf2 signaling pathways in broilers. Toxins.

[182] Rajput S.A., Sun L., Zhang N., Khalil M.M., Gao X., Ling Z., Zhu L., Khan F.A., Zhang J., Qi D. (2017). Ameliorative effects of grapeseed proanthocyanidin extract on growth performance, immune function, antioxidant capacity, biochemical constituents, liver histpathology and aflatoxin residues in broiler exposed to aflatoxin B1. Toxins.

[183] Abu Hafsa S.H., Ibrahim S.A. (2018). Effect of dietary polyphenol-rich grape seed on growth performance, antioxidant capacity and ileal microflora in broiler chicks. J. Anim. Physiol. Anim. Nutr..

[184] Farahat M.H., Abdallah F.M., Ali H.A., Hernandez-Santana A. (2017). Effect of dietary supplementation of grape seed extract on the growth performance, lipid profile, antioxidant status and immune response of broiler chickens. Animal.

[185] Wang M.L., Suo X., Gu J.H., Zhang W.W., Fang Q., Wang X. (2008). Influence of grape seed proanthocyanidin extract in broiler chickens: Effect on chicken coccidiosis and antioxidant status. Poult. Sci..

[186] Yang J.Y., Zhang H.J., Wang J., Wu S.G., Yue H.Y., Jiang X.R., Qi G.H. (2017). Effects of dietary grape proanthocyanidins on the growth performance, jejunum morphology and plasma biochemical indices of broiler chicks. Animal.

[187] Mavrommatis A., Giamouri E., Myrtsi E.D., Evergetis E., Filippi K., Papapostolou H., Koulocheri S.D., Zoidis E., Pappas A.C., Koutinas A. (2021). Antioxidant status of broiler chickens fed diets supplemented with vinification by-products: A valorization approach. Antioxidants.

[188] Mavrommatis A., Simitzis P.E., Kyriakaki P., Giamouri E., Myrtsi E.D., Evergetis E., Filippi K., Papapostolou H., Koulocheri S.D., Pappas A.C. (2021). Immune-related gene expression profiling of broiler chickens fed diets supplemented with vinification byproducts: A valorization approach II. Animals.

[189] Duangnumsawang Y., Zentek J., Vahjen W., Tarradas J., Boroojeni F.G. (2022). Alterations in bacterial metabolites, cytokines, and mucosal integrity in the caecum of broilers caused by feed additives and host-related factors. Front. Physiol..

[190] Duangnumsawang Y., Zentek J., Vahjen W., Tarradas J., Boroojeni F.G. (2023). Impact of feed additives and host-related factors on bacterial metabolites, mucosal integrity and immune response in the ileum of broilers. Vet. Res. Commun..

[191] Brenes A., Viveros A., Goni I., Centeno C., Sayago-Ayerdy S.G., Arija I., Saura-Calixto F. (2008). Effect of grape pomace concentrate and vitamin E on digestibility of polyphenols and antioxidant activity in chickens. Poult. Sci..

[192] Makri S., Kafantaris I., Stagos D., Chamokeridou T., Petrotos K., Gerasopoulos K., Mpesios A., Goutzourelas N., Kokkas S., Goulas P. (2017). Novel feed including bioactive compounds from winery wastes improved broilers’ redox status in blood and tissues of vital organs. Food Chem. Toxicol..

[193] Gungor E., Altop A., Erener G. (2021). Effect of raw and fermented grape pomace on the growth performance, antioxidant status, intestinal morphology, and selected bacterial species in broiler chicks. Animals.

[194] de-Cara A., Saldana B., Vázquez P., Rey A.I. (2023). Dietary protected sodium butyrate and/or olive leaf and grape-based by-product supplementation modifies productive performance, antioxidant status and meat quality in broilers. Antioxidants.

[195] Nardoia M., Romero C., Brenes A., Arija I., Viveros A., Ruiz-Capillas C., Chamorro S. (2020). Addition of fermented and unfermented grape skin in broilers’ diets: Effect on digestion, growth performance, intestinal microbiota and oxidative stability of meat. Animal.

[196] Romero C., Nardoia M., Arija I., Viveros A., Rey A.I., Prodanov M., Chamorro S. (2021). Feeding broiler chickens with grape seed and skin meals to enhance α- and γ-tocopherol content and meat oxidative stability. Antioxidants.

[197] Romero C., Nardoia M., Brenes A., Arija I., Viveros A., Chamorro S. (2021). Combining grape byproducts to maximise biological activity of polyphenols in chickens. Animals.

[198] Turcu R.P., Panaite T.D., Untea A.E., Soica C., Iuga M., Mironeasa S. (2020). Effects of supplementing grape pomace to broilers fed polyunsaturated fatty acids enriched diets on meat quality. Animals.

[199] Aditya S., Ohh S.-J., Ahammed M., Lohakare J. (2018). Supplementation of grape pomace (*Vitis vinifera*) in broiler diets and its effect on growth performance, apparent total tract digestibility of nutrients, blood profile and meat quality. Anim. Nutr..

[200] Bennato F., Di Luca A., Martino C., Ianni A., Marone E., Grotta L., Ramazzotti S., Cichelli A., Martino G. (2020). Influence of grape pomace intake on nutritional value, lipid oxidation and volatile profile of poultry meat. Foods.

[201] Tufarelli V., Baghban-Kanani P., Azimi-Youvalari S., Hosseintabar-Ghasemabad B., Slozhenkina M., Gorlov I., Viktoronova F.M., Seidavi A., Laudadio V. (2022). Effect of dietary flaxseed meal supplemented with dried tomato and grape pomace on performance traits and antioxidant status of laying hens. Anim. Biotechnol..

[202] Selim S., Abdel-Megeid N.S., Alhotan R.A., Ebrahim A., Hussein E. (2023). Grape pomace: Agrifood by-product with potential to enhance performance, yolk quality, antioxidant capacity, and eggshell ultrastructure in laying hens. Vet. Sci..

[203] Reis J.H., Gebert R.R., Barreta M., Boiago M.M., Souza C.F., Baldissera M.D., Santos I.D., Wagner R., Laporta L.V., Stefani L.M. (2019). Addition of grape pomace flour in the diet on laying hens in heat stress: Impacts on health and performance as well as the fatty acid profile and total antioxidant capacity in the egg. Therm. Biol..

[204] Herranz B., Romero C., Sanchez-Roman I., Lopez-Torres M., Viveros A., Arija I., Alvarez M.D., de Pascual-Teresa S., Chamorro S. (2024). Enriching eggs with bioactive compounds through the inclusion of grape pomace in laying hens diet: Effect on internal and external egg quality parameters. Foods.

[205] Hafeez A., Hassni S.F., Naz S., Alonaizan R., Al-Akeel R.K., Sifa D., Shamsi S., Khan R.U. (2023). Impact of grape (*Vitis vinifera*) seed extract on egg production traits, nutrient digestibility, lipid peroxidation and fertility of golden hens (*Gallus gallus*) during early stage production. Vet Q..

[206] Hafeez A., Ullah S., Naz S., Alrefaei A.F., Khan R.U., Abdelrahman S.H., Losacco C., Selvaggi M. (2024). Effect of dietary polyphenol rich grape (*Vitis vinifera*) seed extract supplementation on production performance, egg quality, plasma MDA, reproductive performance and faecal microbiota of golden laying hens. J. Appl. Anim. Res..

[207] Kaya A., Yildirim B.A., Kaya H., Gül M., Celebi S. (2014). The effects of diets supplemented with crushed and extracted grape seed on performance, egg quality parameters, yolk peroxidation and serum traits in laying hens. Eur. Poult. Sci..

[208] Kara K., Güclü B.K., Baytok E., Sentürk M. (2016). Effects of grape pomace supplementation to laying hen diet on performance, egg quality, egg lipid peroxidation and some biochemical parameters. J. Appl. Anim. Res..

[209] Romero C., Arija I., Viveros A., Chamorro S. (2022). Productive performance, egg quality and yolk lipid peroxidation in laying hens fed diets including grape pomace or grape extract. Animals.

[210] Grigorova S., Gjorgovska N., Todorova M., Levkov V. (2023). Use of grape marc flour supplementation in laying hens’ diet on laying productivity, egg quality and biochemical parameters. Vet. Zootechnika.

[211] Akter A., Li X., Grey E., Wang S.C., Kebreab E. (2025). Grape pomace supplementation reduced methane emissions and improvedmilk quality in lactating dairy cows. J. Dairy Sci..

[212] Moate P.J., Jacobs J.L., Hixson J.L., Deighton M.H., Hannah M.C., Morris G.L., Ribaux B.E., Wales W.J., Williams S.R.O. (2020). Effects of feeding either red or white grape marc on milk production and methane emissions from early-lactation dairy cows. Animals.

[213] Moate P.J., Williams S.R., Torok V.A., Hannah M.C., Ribaux B.E., Tavendale M.H., Eckard R.J., Jacobs J.L., Auldist M.J., Wales W.J. (2014). Grape marc reduces methane emissions when fed to dairy cows. J. Dairy Sci..

[214] Chedea V.S., Pelmus R.S., Lazar C., Pistol G.C., Calin L.G., Toma S.M., Dragomir C., Taranu I. (2017). Effects of a diet containing dried grape pomace on blood metabolites and milk composition of dairy cows. J. Sci. Food Agric..

[215] Ianni A., Di Maio G., Pittia P., Grotta L., Perpetuini G., Tofalo R., Cichelli A., Martino G. (2019). Chemical–nutritional quality and oxidative stability of milk and dairy products obtained from Friesian cows fed with a dietary supplementation of dried grape pomace. J. Sci. Food Agric..

[216] Ianni A., Innosa D., Martino C., Bennato F., Martino G. (2019). Compositional characteristics and aromatic profile of caciotta cheese obtained from Friesian cows fed with a dietary supplementation of dried grape pomace. J. Dairy Sci..

[217] Ianni A., Martino G. (2020). Dietary grape pomace supplementation in dairy cows: Effect on nutritional quality of milk and its derived dairy products. Foods.

[218] Manso T., Gallardo B., Salvá A., Guerra-Rivas C., Mantecón A.R., Lavín P., De la Fuente M.A. (2016). Influence of dietary grape pomace combined with linseed oil on fatty acid profile and milk composition. J. Dairy Sci..

[219] Kang D., Lungu S.E., Danso F., Dzou C.F., Chen Y., Zheng X., Nie F., Lin H., Chen J., Zhou G. (2025). Animal health and nutrition: Metabolic disorders in cattle and improvement strategies. Front. Vet. Sci..

[220] Tufarelli V., Puvaca N., Glamocic D., Pugliese G., Colonna M.A. (2024). The most important metabolic diseases in dairy cattle during the transition period. Animals.

[221] Gross J.J., Schwarz F.J., Eder K., van Dorland H.A., Bruckmaier R.M. (2013). Liver fat content and lipid metabolism in dairy cows during early lactation and during a mid-lactation feed restriction. J. Dairy Sci..

[222] Plaizier J.C., Krause D.O., Gozho G.N., McBride B.W. (2008). Subacute ruminal acidosis in dairy cows: The physiological causes, incidence and consequences. Vet. J..

[223] Vels L., Rontved C.M., Bjerring M., Ingvartsen K.L. (2009). Cytokine and acute phase protein gene expression in repeated liver biopsies of dairy cows with a lipopolysaccharide-induced mastitis. J. Dairy Sci..

[224] Zebeli Q., Metzler-Zebeli B.U., Ametaj B.N. (2012). Meta-analysis reveals threshold level of rapidly fermentable dietary concentrate that triggers systemic inflammation in cattle. J. Dairy Sci..

[225] Bionaz M., Trevisi E., Calamari L., Librandi F., Ferrari A., Bertoni G. (2007). Plasma paraoxonase, health, inflammatory conditions, and liver function in transition dairy cows. J. Dairy Sci..

[226] Gessner D.K., Schlegel G., Keller J., Schwarz F.J., Ringseis R., Eder K. (2013). Expression of target genes of nuclear factor E2-related factor 2 in the liver of dairy cows in the transition period and at different stages of lactation. J. Dairy Sci..

[227] Abuelo A., Hernandez J., Benedito J.L., Castillo C. (2019). Redox biology in transition periods of dairy cattle: Role in the health of periparturient and neonatal animals. Antioxidants.

[228] Yonekura S. (2024). The role of endoplasmic reticulum stress in metabolic diseases and mammary epithelial cell homeostasis in dairy cows. Anim. Sci. J..

[229] Gessner D.K., Koch C., Romberg F.J., Winkler A., Dusel G., Herzog E., Most E., Eder K. (2015). The effect of grape seed and grape marc meal extract on milk performance and the expression of genes of endoplasmic reticulum stress and inflammation in the liver of dairy cows in early lactation. J. Dairy Sci..

[230] Kim K.H., Lee M.S. (2014). FGF21 as a stress hormone: The roles of FGF21 in stress adaptation and the treatment of metabolic diseases. Diabetes Metab. J..

[231] Gessner D.K., Winkler A., Koch C., Dusel G., Liebisch G., Ringseis R., Eder K. (2017). Analysis of hepatic transcript profile and plasma lipid profile in early lactating dairy cows fed grape seed and grape marc meal extract. BMC Genom..

[232] Gobert M., Martin B., Ferlay A., Chilliard Y., Graulet B., Pradel P., Bauchart D., Durand D. (2009). Plant polyphenols associated with vitamin E can reduce plasma lipoperoxidation in dairy cows given n-3 polyunsaturated fatty acids. J. Dairy Sci..

[233] Signor M.H., de Freitas Dos Santos A.L., de Vitt M.G., Nora L., Lago R.V.P., Wolschick G.J., Correa N.G., Klein B., Xavier A.C.H., Wagner R. (2024). Grape seed oil in the diet of primiparous Jersey cows before and after parturition: Effects on performance, health, rumen environment, and milk quality. Trop. Anim. Health Prod..

[234] Huang Y., Oikonomou G., Hu J., Li Y., Du X., Du Y., Liu Y., Zhang P., Wang P., Yu H. (2019). Effect of feeding grape seed Proanthocyanidin extract on production performance, metabolic and anti-oxidative status of dairy cattle. Arq. Bras. Med. Vet. Zootec..

[235] Urkmez E., Biricik H. (2022). Grape seed extract supplementation in heat-stressed preweaning dairy calves: I. Effects on antioxidant status, inflammatory response and physiological parameters. Anim. Feed Sci. Technol..

[236] Ma J., Fan X., Zhang W., Zhou G., Yin F., Zhao Z., Gan S. (2023). Grape seed extract as a feed additive improves the growth performance, ruminal fermentation and immunity of weaned beef calves. Animals.

[237] Iannaccone M., Elgendy R., Giantin M., Martino C., Giansante D., Ianni A., Dacasto M., Martino G. (2018). RNA sequencing-based whole transcriptome analysis of Friesian cattle fed with grape pomace-supplemented diet. Animals.

[238] Molosse V.L., Deolindo G.L., Lago R.V.P., Cecere B.G.O., Zotti C.A., Vedovato M., Copetti P.M., Fracasso M., Morsch V.M., Xavier A.C. (2023). The effects of the inclusion of ensiled and dehydrated grape pomace in beef cattle diet: Growth performance, health and economic viability. Anim. Sci. Technol..

[239] Li Y., Shi C., Deng J., Qiu X., Zhang S., Wang H., Qin X., He Y., Cao B., Su H. (2024). Effects of grape pomace on growth performance, nitrogen metabolism, antioxidants, and microbial diversity in Angus bulls. Antioxidants.

[240] Korver D.R. (2023). Review: Current challenges in poultry nutrition, health, and welfare. Animal.

[241] Durand D., Collin A., Merlot E., Baeza E., Guilloteau L.A., Le Floc’h N., Thomas A., Fontagne-Dicharry S., Gondret F. (2022). Review: Implications of redox balance in animal health and performance at critical periodsm insights from different farm animal species. Animal.

[242] Horst E.A., Mayorga E.J., Baumgard L.H. (2025). International Symposium on Ruminant Physiology: Integrating our understanding of stress physiology. J. Dairy Sci..

[243] Faria A., Fernandes I., Norberto S., Mateus N., Calhau C. (2014). Interplay between anthocyans and gut microbiota. J. Agricult. Food Chem..

[244] Cardona F., Andrés-Lacueva C., Tulipani S., Tinahones F.J., Queipo-Ortuno M.I. (2013). Benefits of polyphenols on gut microbiota and implications in human health. J. Nutr. Biochem..

[245] Yang Y., Sun Y., Gu T., Yan Y., Guo J., Zhang X., Pang H., Chen J. (2025). The metabolic characteristics and bioavailability of resveratrol based on metabolic enzymes. Nutr. Rev..

[246] Cassidy A., Minihane A.-M. (2017). The role of metabolism (and the microbiome) in defining the clinical efficacy of dietary flavonoids. Am. J. Clin. Nutr..

[247] Rogowska-van der Molen M.A., Berasategui-Lopez A., Coolen S., Jansen R.S., Welte C.U. (2023). Microbial degradation of plant toxins. Microbiol..

[248] Scott M.B., Styring A.K., McCullagh J.S.O. (2022). Polyphenols: Bioavailability, microbiome interactions and cellular effects on health in humans and animals. Pathogens.

[249] Chamorro S., Romero C., Brenes A., Sanchez-Patan F., Bartolome B., Viveros A., Arija I. (2019). Impact of a sustained consumption of grape extract on digestion, gut microbial metabolism and intestinal barrier in broiler chickens. Food Funct..

[250] Anghel A.C., Taranu I., Ortan A., Marcu Spinu S., Dragoi Cudalbeanu M., Rosu P.M., Babeanu N.E. (2024). Polyphenols and microbiota modulation: Insights from swine and other animal models for human therapeutic strategies. Molecules.

[251] Beaumont M., Lencina C., Painteaux L., Viemon-Desplanque J., Phornlaphat O., Lambert W., Chalvon-Demersay T. (2022). A mix of functional amino acids and grape polyphenols promotes the growth of piglets, modulates the gut microbiota in vivo and regulates epithelial homeostasis in intestinal organoids. Amino Acids.

[252] Lu Y., Zhao M., Mo J., Lan G., Liang J. (2022). Dietary supplementation ellagic acid on the growth, microbiota, and inflammation in weaned pigs. Front. Vet. Sci..

[253] Ringseis R., Gessner D.K., Eder K. (2020). The gut-liver axis in the control of energy metabolism and food intake in animals. Annu. Rev. Anim. Biosci..

[254] Wang J., Tong T., Yu C., Wu Q. (2025). The research progress on the impact of gut microbiota on health and production performance. Front. Vet. Sci..

[255] Baron C.P., Andersen J.J. (2017). Myoglobin-induced lipid peroxidation. J. Agric. Food Chem..

[256] Ghani M.A., Barril C., Bedgood D.R., Prenzler P.D. (2017). Measurement of antioxidant activity with the thiobarbituric acid reactive substances assay. Food Chem..

[257] Sevanian A., McLeod L.L. (1987). Cholesterol autoxidation in phospholipid membrane bilayers. Lipids.

[258] Brandsch C., Ringseis R., Eder K. (2002). High dietary iron concentrations enhance the formation of cholesterol oxidation products in the liver of adult rats fed salmon oil with minimal effects on antioxidant status. J. Nutr..

[259] Gentile C.L., Frye M., Pagliassotti M.J. (2011). Endoplasmic reticulum stress and the unfolded protein response in nonalcoholic fatty liver disease. Antioxid. Redox Signal..

[260] Kolattukudy P.E., Niu J. (2012). Inflammation, endoplasmic reticulum stress, autophagy, and the monocyte chemoattractant protein-1/CCR2 pathway. Circ. Res..

[261] Jefferi N.E.S., Shamhari A.A., Hamid Z.A., Budin S.B., Taib I.S. (2025). Interlinkage between inflammation, oxidative stress, and endoplasmic reticulum stress in bisphenol-induced testicular steroidogenesis disturbance: A mini review. Int. J. Reprod. Biomed..

[262] Tang Y., Zhou X., Cao T., Chen E., Li Y., Lei W., Hu Y., He B., Liu S. (2022). Endoplasmic reticulum stress and oxidative stress in inflammatory diseases. Cell Biol..

[263] Ajoolabady A., Kaplowitz N., Lebeaupin C., Kroemer G., Kaufman R.J., Malhi H., Ren J. (2023). Endoplasmic reticulum stress in liver diseases. Hepatology.

[264] He Z., Liu Q., Wang Y., Zhao B., Zhang L., Yang X., Wang Z. (2025). The role of endoplasmic reticulum stress in type 2 diabetes mellitus mechanisms and impact of islet function. PeerJ.

[265] Li M., Zhao B., Wang J., Zhang H., Yang Y., Song S., Psifidi A., Wu W., Loor J.J., Xu C. (2025). Caveolin 1 in bovine liver is associated with fatty acid-induced lipid accumulation and the endoplasmic reticulum unfolded protein response: Role in fatty liver development. J. Dairy Sci..

[266] Sharmin M.M., Mizusawa M., Hayashi S., Arai W., Sakata S., Yonekura S. (2020). Effects of fatty acids on inducing endoplasmic reticulum stress in bovine mammary epithelial cells. J. Dairy Sci..

[267] Li C., Huang J., Chen X., Yan Y., Li L., Zhao W. (2022). Transcriptome analysis reveals that NEFA and β-hydroxybutyrate induce oxidative stress and inflammatory response in bovine mammary epithelial cells. Metabolites.

[268] Shen T., Xia S., Usman M., Xu X., Loor J.J., Xu C. (2025). Nuclear factor erythroid 2-related factor 1 regulates the expression of proteosomal genes in ketotic kows and protects mammary cells against free fatty acid-induced endoplasmic reticulum stress. J. Dairy Sci..

[269] Zhang H., Chen Y., Li Y., Jia P., Ji S., Chen Y., Wang T. (2020). Protective effects of pterostilbene against damage, redox imbalance, mitochondrial dysfunction, and endoplasmic reticulum stress in weanling pigs. J. Anim. Sci..

[270] Gessner D.K., Gröne B., Rosenbaum S., Most E., Hillen S., Becker S., Erhardt G., Reiner G., Ringseis R., Eder K. (2015). Effect of a negative energy balance induced by feed restriction on pro-inflammatory and endoplasmic reticulum stress signalling pathways in the liver and skeletal muscle of lactating sows. Arch. Anim. Nutr..

[271] Gessner D.K., Gröne B., Couturier A., Rosenbaum S., Hillen S., Becker S., Erhardt G., Reiner G., Ringseis R., Eder K. (2015). Dietary Fish Oil Inhibits Pro-Inflammatory and ER Stress Signalling Pathways in the Liver of Sows during Lactation. PLoS ONE.

[272] Jing J., Zeng H., Shao Q., Tang J., Wang L., Jia G., Liu G., Chen X., Tian G., Cai J. (2023). Selenomethionine alleviates environmental heat stress induced hepatic lipid accumulation and glycogen infiltration of broilers via maintaining mitochondrial and endoplasmic reticulum homeostasis. Redox Biol..

[273] Brito M.L., Coutinho-Wolino K.S., Almeida P.P., Trigueira P.C., Alves A.P.P., Magliano D.C., Stockler-Pinto M.B. (2024). Unstressing the reticulum: Nutritional strategies for modulating endoplasmic reticulum stress in obesity. Mol. Nutr. Food Res..

[274] Wei H., Li H., Miao D., Wang H., Liu Y., Xing L., Bao J., Li J. (2024). Dietary resveratrol supplementation alleviates cold exposure-induced pyroptosis and inflammation in broiler heart by modulating oxidative stress and endoplasmic reticulum stress. Poult Sci..

[275] Fu K., Chen L., Miao L., Guo Y., Zhang W., Bai Y. (2019). Grape seed proanthocyanidins protect N2a cells against ischemic injury via endoplasmic reticulum stress and mitochondrial-associated pathways. Neurol. Disord. Drug Targets.

[276] Chamorro S., Viveros A., Centeno C., Romero C., Arija I., Brenes A. (2013). Effects of dietary grape seed extract on growth performance, amino acid digestibility and plasma lipids and mineral content in broiler chicks. Animal.

[277] Grosu I.A., Pistol G.C., Marin D.E., Cismileanu A., Palade L.M., Taranu I. (2020). Effects of dietary grape seed meal bioactive compounds on the colonic microbiota of weaned piglets with dextran sodium sulfate-induced colitis used as an inflammatory model. Front. Vet. Sci..

[278] Chamorro S., Viveros A., Rebolé A., Rica B.D., Arija I., Brenes A. (2015). Influence of dietary enzyme addition on polyphenol utilization and meat lipid oxidation of chicks fed grape pomace. Int. Food Res. J..

[279] Ebrahimzadeh S., Navidshad B., Farhoomand P., Aghjehgheshlagh F.M. (2018). Effects of exogenous tannase enzyme on growth performance, antioxidant status, immune response, gut morphology and intestinal microflora of chicks fed grape pomace. S. Afr. J. Anim. Sci..

[280] Jonathan O., Mnisi C.M. (2021). Effect of dietary red grape pomace on growth performance, hematology, serum biochemistry, and meat quality parameters in Hy-line Silver Brown cockerels. PLoS ONE.

[281] Pascariu S., Pop I., Simeanu D., Pavel G., Solcan C. (2017). Effects of wine by-products on growth performance, complete blood count and total antioxidant status in broilers. Braz. J. Poult. Sci..

[282] Afsana K., Shiga K., Ishizuka S., Hara H. (2004). Reducing effect of ingesting tannic acid on the absorption of iron, but not of zinc, copper and manganese by rats. Biosci. Biotechnol. Biochem..

[283] Gaffney S., Williams V., Flynn P., Carlino R., Mowry C., Dierenfeld E., Babb C., Fan J., Tramontano W.A. (2004). Tannin/Polyphenol effects on iron solubilization in vitro. Bios.

[284] Fiesel A., Ehrmann M., Gessner D.K., Most E., Eder K. (2015). Effects of polyphenol-rich plant products from grape or hop as feed supplements on iron, zinc and copper status in piglets. Arch. Anim. Nutr..

[285] Thanh L.P., Kha P.T.T., Loor J.J., Hang T.T.T. (2022). Grape seed tannin extract and polyunsaturated fatty acids affect in vitro ruminal fermentation and methane production. J. Anim. Sci..

